# A novel GAM-based method for predicting phosphate (PO_4_) concentrations in irrigated drainage/return-flow systems

**DOI:** 10.7717/peerj.21181

**Published:** 2026-04-29

**Authors:** Müge Erkan Can

**Affiliations:** Department of Agricultural Structures and Irrigation, Faculty of Agriculture, Çukurova University, Adana, Türkiye

**Keywords:** Phosphate prediction, Generalized additive model (GAM), Water quality monitoring, Agricultural pollution, Machine learning

## Abstract

Phosphate (PO_4_) pollution in irrigated catchments and their return-flow and drainage networks threatens water quality and agricultural sustainability, particularly under conditions of intensive fertilization and shallow groundwater. This study presents a predictive approach to estimate PO_4_ concentration using a Generalized Additive Model (GAM) based on daily monitoring data from the Akarsu Irrigation District in Türkiye’s Lower Seyhan Plain. Here, the modeled variable is PO_4_ in irrigation return-flow/drainage water, measured at the main drainage outlet (L4), which integrates excess irrigation water that has passed through the agricultural landscape and collected surface runoff and subsurface drainage. Downstream of L4, drainage water is conveyed by the main drainage channel; part is reused for irrigation, and the remainder flows toward lagoon and wetland areas and ultimately the Mediterranean Sea. The dataset comprised 522 daily observations from the 2022–2023 water years and included nitrate (NO_3_), nitrite (NO_2_), electrical conductivity (EC), pH (hydrogen ion activity), flow rate (Q), and precipitation (P) as predictors. Despite weak pairwise correlations of PO_4_ with individual variables (maximum *r* = 0.1293 with NO_3_), the GAM captured nonlinear multivariate relationships and produced good agreement between predicted and measured PO_4_ at the L4 outlet (mean squared error (MSE) = 0.019966; root mean squared error (RMSE) = 0.1413 mg L^−1^; mean error = −0.00457 mg L^−1^; error SD = 0.14136 mg L^−1^), indicating minimal bias and stable performance. In benchmark comparisons using identical inputs and the same time-structured validation design (80/20 split; random splits were used only for sensitivity analysis), the GAM substantially outperformed linear regression (LR), artificial neural network (ANN), and support vector machine (SVM), which showed very low predictive skill (R^2^ ≈ 0.03–0.05). Predictive performance was evaluated primarily using error-based metrics; R^2^ was reported only as a goodness-of-fit measure. The L4 outlet drains an intensively managed agricultural catchment dominated by irrigated cropland. Model fit, expressed as explained variance values (training R^2^ = 0.832; testing R^2^ = 0.788), indicated consistent performance without evidence of substantial overfitting. Overall, the findings demonstrate that GAM-based estimation can reliably reproduce both peak and moderate PO_4_ concentrations and serve as a practical screening tool for nutrient monitoring at irrigated drainage/return-flow outlets. By leveraging routinely monitored variables, the model can reduce the frequency of laboratory PO_4_ assays—often requiring additional reagents, consumables, and handling time—thereby lowering analytical workload and spectrophotometric operating time while enabling near-real-time assessment of PO_4_ dynamics. These results support the use of data-driven estimation to inform nutrient management and reduce eutrophication risk in irrigated catchments by monitoring drainage exports.

**Background:**

Phosphate pollution in irrigation areas, particularly in regions with shallow groundwater and intensive agriculture, poses serious environmental and agricultural risks, including eutrophication and water-quality degradation. Conventional methods for phosphate monitoring are often time-consuming, costly, and spatially limited, making them unsuitable for real-time applications. Furthermore, the complex, nonlinear interactions between phosphate concentrations and environmental variables, including nitrate, nitrite, pH, EC, flow rate, and precipitation, challenge traditional predictive approaches. While various machine learning models have been explored for phosphorus prediction, their computational demands and overfitting risks often limit their field-level applicability. Therefore, this study aimed to develop a robust, efficient, and interpretable method for predicting phosphate concentrations using a GAM and leveraging daily environmental data collected in a Mediterranean irrigation district in Türkiye.

**Methods:**

Daily water samples were collected at the outlet of the L4 agricultural catchment in the Akarsu Irrigation District (AID) on the Lower Seyhan Plain, Türkiye, during the 2022 and 2023 water years. The area is characterized by intensively managed irrigated cropland and shallow groundwater conditions. A total of 522 daily observations were compiled, including PO_4_, NO_3_, NO_2_, EC, pH, flow rate (Q), and precipitation (P). Laboratory analyses were performed using spectrophotometric methods for nutrients and electrochemical measurements of EC and pH, while discharge data were obtained from an on-site automatic monitoring and sampling system.

A GAM was developed to represent nonlinear relationships between PO_4_ and the predictor variables using penalized smoothing functions. Because the dataset is a daily time series, temporal dependence was addressed by including a smoother for time (date/time index) and by fitting the model with an AR(1) residual correlation structure (GAMM). To ensure realistic model evaluation under temporal dependence, predictive skill was assessed primarily using a time-structured (blocked, contiguous) 80/20 split, with the earlier 80% of observations used for training and the later 20% for testing. To assess robustness to the choice of partition (sensitivity analysis only), we additionally repeated the split–fit–evaluate procedure over 100 independent randomized 80/20 splits. These random-split results are reported as a secondary check and are not interpreted as the main estimate of predictive skill under autocorrelation. Model predictive performance was primarily assessed using error-based metrics (MSE, RMSE, bias/mean error, and error SD), while R^2^ was reported only as explained variance (goodness-of-fit). Residual diagnostics, including inspection of the residual distribution and autocorrelation (ACF), were used to evaluate model assumptions, stability, and potential overfitting.

**Results:**

This study developed a data-driven method for estimating PO_4_ concentrations at the L4 drainage outlet using a Generalized Additive Model. Although same-day Pearson correlations between PO_4_ and routinely monitored predictors (EC, pH, Q, P, NO_2_, NO_3_) were weak (maximum r = 0.1293 for NO_3_), the GAM captured nonlinear and conditional multivariate effects. It demonstrated strong agreement between predicted and measured PO_4_ values. Model performance was evaluated primarily using error-based metrics, yielding MSE = 0.019966; RMSE = 0.1413 mg L^−1^; mean error (bias) = −0.00457 mg L^−1^; and error SD = 0.14136 mg L^−1^. R^2^ was reported only as explained variance (goodness-of-fit): training R^2^ = 0.8319; testing R^2^ = 0.7875. Because the dataset is a daily time series, temporal dependence was addressed by fitting a GAMM with a smooth function of time and an AR(1) residual structure; and generalization was assessed using a time-structured (blocked or contiguous) train–test split to reduce information leakage from autocorrelation. Repeated random 80/20 splits were used only as a sensitivity analysis and showed consistent performance (mean R^2^ = 0.772, SD = 0.0166 across 100 trials). In benchmark comparisons, the GAM substantially outperformed traditional alternatives (LR, ANN, SVM), which showed very low predictive skill for PO_4_ (R^2^ ≈ 0.03–0.05), highlighting the need for a flexible nonlinear structure to reproduce the observed phosphate dynamics. The model reproduced the overall temporal pattern of PO_4_, while some underestimation remained for the highest short-duration peaks—consistent with the sparse nature of extreme events in the dataset. Overall, the results support the use of the proposed GAM/GAMM framework as an outlet-scale screening tool for near-real-time identification of periods with elevated PO_4_, thereby helping to prioritize laboratory sampling and monitoring efforts when direct PO_4_ measurements are costly or intermittent.

## Introduction

Phosphorus, primarily present as phosphate (PO_4_), is essential for agricultural productivity because it supports plant growth and key physiological functions. In irrigated agricultural landscapes, however, phosphorus applied to fields is typically retained in soils through strong sorption to mineral surfaces and incorporation into organic pools. Elevated PO_4_ levels in irrigation and drainage waters can nevertheless occur when hydrological and transport processes mobilize phosphorus from soil and fertilizers, for example, through surface runoff, erosion and particulate transport, preferential or subsurface flow (including drainage pathways), and sediment–water exchange. These processes can increase nutrient delivery to receiving waters, thereby contributing to eutrophication, algal blooms, and habitat degradation (Carpenter et al., 1998).

In the Akarsu Irrigation District (AID), which covers 9,495 hectares of the Lower Seyhan Plain (LSP) in Türkiye (Alsenjar et al., 2023a; Alsenjar et al., 2023b; Cetin et al., 2020), excess irrigation water from the fields is collected *via* an open drainage network; the monitoring point L4 serves as the main drainage outlet. Therefore, the modeled PO_4_ signal in this study reflects irrigation return-flow/drainage discharge—an integrated mixture of surface runoff and subsurface drainage after water has interacted with soils, fertilizers, and field management practices—rather than the quality of irrigation supply water.

In practical terms, monitoring at L4 serves as an outlet-scale management indicator of nutrient export from the irrigated catchment. In this study, ‘decision support’ refers explicitly to screening-level identification of high-PO_4_ periods to prioritize laboratory sampling, to evaluate operational timing (fertilizer, irrigation, and drainage), and to provide risk-based guidance on drainage water reuse and downstream protection. After passing L4, the drainage/return flow discharge is conveyed through the main drainage channel; downstream farmers reuse a portion of this water for supplemental irrigation, while the remainder continues toward lagoon and wetland areas and ultimately discharges into the Mediterranean Sea. Therefore, PO_4_ information at L4 is relevant not only as a diagnostic signal but also for operational decision support, including identifying high-export periods, prioritizing laboratory sampling and mitigation actions, and providing risk-based guidance for drainage-water reuse and downstream receiving-water protection. Accordingly, prediction is framed as a screening tool to support these actions by enabling near-real-time identification of elevated PO_4_ concentrations and reducing the required frequency of laboratory assays.

Approximately 8 million tons of phosphorus enter aquatic ecosystems each year (Cordell, Drangert & White, 2009). A significant portion of applied phosphate fertilizers accumulates in the soil. Over time, hydrological processes such as surface runoff and subsurface flow can mobilize phosphorus from soils into surrounding aquatic environments—including rivers, lakes, and groundwater—thereby contributing to surface water eutrophication and subsurface phosphorus pollution. As phosphorus levels rise in shallow groundwater, particularly in riparian zones adjacent to water bodies, phosphorus can significantly influence the total phosphorus flux to surface-water systems during groundwater recharge (Kazmierczak et al., 2016; Yu et al., 2018). In this context, timely information on PO_4_ dynamics at catchment outlets (*e.g.*, drainage and return flows) supports practical management actions—such as identifying high-export events, prioritizing sampling and mitigation, and evaluating the effectiveness of nutrient and drainage management. Therefore, PO_4_ prediction is used as an operational decision-support and screening tool, not as a direct control mechanism, to reduce monitoring effort and costs by decreasing the frequency of laboratory PO_4_ assays and to enable rapid detection of periods with elevated concentrations and loads. This capability is particularly relevant in irrigated landscapes where water quality conditions can change rapidly.

Although modeling techniques have advanced, predicting PO_4_ concentrations in aquatic systems remains complex because nonlinear interactions among hydrological conditions, biogeochemical processes, and catchment management practices can produce rapid temporal variability at monitoring points (Schoumans et al., 2014). Conventional statistical approaches, such as linear regression, often fail to capture these complexities because they rely on assumptions of linearity and homogeneity that rarely hold for environmental datasets (Helsel et al., 2020). In irrigation and drainage networks, PO_4_ is commonly quantified using laboratory-based analyses that require sampling, reagents and consumables, and instrument time (*e.g.*, spectrophotometry). Even at a single outlet location, high-frequency monitoring can be logistically demanding and costly when sustained over long periods. Therefore, establishing reliable and efficient predictive models based on routinely measured variables can support screening-level, near-real-time estimation of PO_4_ at drainage outlets and strengthen data-driven irrigation–drainage management by enabling outlet-scale nutrient-export screening and load-relevant monitoring (concentration × discharge).

Phosphorus is a core nutrient that supports energy transfer, photosynthesis, and root development. Maintaining appropriate PO_4_ levels is crucial for maximizing crop yield. However, elevated PO_4_ concentrations in runoff and drainage water can occur when multiple phosphorus sources—including agricultural inputs (fertilizer application and legacy soil P) and, where present, domestic or municipal discharges or other external inputs—coincide with hydrological transport pathways such as runoff, erosion, and drainage return flows. These conditions can increase phosphorus delivery to receiving waters and may contribute to eutrophication risk and, in some settings, to shallow groundwater enrichment (Tobia & Milad, 1964). Accordingly, this study proposes PO_4_ forecasting at the drainage-outlet scale as a monitoring and decision-support tool to track temporal variability, identify high-export periods, and support targeted sampling and evaluation of management actions (*e.g.*, fertilizer timing, irrigation and drainage operations, and mitigation measures), without implying that agriculture is necessarily the dominant source in all systems.

Despite the availability of various modelling approaches, predicting PO_4_ remains challenging because relationships between PO_4_ concentrations and their drivers are often nonlinear (Withers et al., 2014). A substantial body of literature addresses the multiple facets of the phosphorus cycle (Schilling et al., 2020), including the identification of phosphorus sources (Warrack, Kang & Von Sperber, 2021), the conditions driving phosphorus transformation (Mabilde, De Neve & Sleutel, 2017), the mechanisms of phosphorus retention and release in soils (Flower et al., 2016), and assessments of pollution loads (Meinikmann, Hupfer & Lewandowski, 2015). Much research has focused on phosphorus dynamics in surface waters, such as rivers and lakes (Shen et al., 2019; Hanson et al., 2020; Cajucom et al., 2024). However, reliable prediction of phosphorus concentrations in groundwater and drainage waters remains limited, since most groundwater pollution models primarily focus on nitrate (NO_3_-N) contamination (Yang et al., 2023; Veeramanju, 2024).

Water quality assessment is therefore central to environmental protection, field management, and pollution control, yet budgetary constraints—particularly in developing countries—often prevent comprehensive monitoring. Manual sampling and laboratory analysis remain core methods for PO_4_ determination (Hussein et al., 2024), but while accurate, they are constrained by limited spatial and temporal coverage, and by high costs and labor requirements, rendering them suboptimal for large-scale or near-real-time irrigation management. Moreover, traditional linear statistical models struggle to capture the inherent unpredictability of environmental conditions, soil properties, and agricultural practices; assumptions of linearity and homogeneity can result in inadequate representation of PO_4_ behaviour in irrigation systems (Huang et al., 2017).

On the other hand, machine learning and advanced statistical approaches, such as Generalized Additive Models (GAMs), have shown considerable potential in overcoming these constraints. GAMs are particularly well-suited to modeling complex, non-linear relationships and can effectively handle heterogeneous and fragmented datasets (Wood, 2017). Their flexibility allows the underlying patterns of the data to be represented without assuming a fixed functional form between the predictors and the response variable (Guisan, Edwards Jr & Hastie, 2002; Gomez-Rubio, 2018). This characteristic makes GAMs especially valuable in environmental studies, where variability is high, and relationships among variables are rarely linear. Nevertheless, GAM performance depends on the (quasi-)stationarity of the predictor–PO_4_ relationship during deployment. If system conditions shift—due to changes in agricultural practices, hydrological regime, source composition, or seasonal operations—predictive skill may degrade, and the model should be updated or retrained using newly observed PO_4_ measurements. Therefore, GAM-based estimation is best framed as a decision-support and screening approach—supported by periodic ground-truth sampling and routine performance checks (*e.g.*, tracking RMSE and bias), which trigger recalibration when needed—that reduces—but does not eliminate—laboratory monitoring.

In addition, GAMs can be combined with techniques such as Principal Component Analysis (PCA) to address multicollinearity, a frequent issue in environmental datasets (Ispany et al., 2018). Because PCA is a linear transformation, it should be applied cautiously and primarily to reduce redundancy among highly correlated predictors; in strongly nonlinear settings, PCA may distort the geometry of predictors or distribute nonlinear signals across multiple components. Accordingly, any PCA-based truncation should be justified by demonstrating that predictive information is retained (*e.g.*,  *via* cross-validated performance), and alternative strategies—such as correlation-based screening, penalized term selection/shrinkage within GAM, or nonlinear/regularized dimension-reduction methods—may be preferable when nonlinear structure is critical. They require minimal parameter adjustment and are computationally more efficient than many machine learning algorithms, which often demand extensive hyperparameter tuning. For example, in corrosion prediction tasks, GAM-based models have outperformed neural networks such as Backpropagation Neural Networks (BPNN) and Generalized Regression Neural Networks (GRNN), demonstrating both higher predictive accuracy and faster computation times (Zhang & Yang, 2023). These properties make GAMs attractive for rapid, interpretable screening and decision-support applications, provided that model performance is monitored and updated when system conditions change.

Furthermore, hybrid models that integrate GAMs with other machine learning techniques have successfully estimated nutrient concentrations in watershed environments, demonstrating their utility in complex environmental contexts (Fang, Deitch & Gebremicael, 2025). As non-parametric methods, GAMs reduce the need for extensive parameter calibration, shorten model development time, and lower computational demands (Wood, 2017). Their flexibility allows the underlying patterns of the data to be represented without assuming a fixed functional form between the predictors and the response variable (Guisan, Edwards Jr & Hastie, 2002; Gomez-Rubio, 2018). In this study, the relevance of GAM is explicitly framed in the context of irrigation, return-flow, and drainage monitoring, where PO_4_ dynamics at outlet points reflect nonlinear interactions among hydrology, management practices, and water-quality processes (Drexler & Ainsworth, 2013).

GAMs provide a versatile framework for simulating non-linear relationships between target pollutants and environmental variables. They can therefore support outlet-scale assessment of irrigation return flows, in which datasets may be heterogeneous and influenced by multiple interrelated drivers (Drexler & Ainsworth, 2013). To avoid over-generalisation, the methodological context is restricted to irrigation, drainage, and water-quality applications relevant to nutrient dynamics, rather than listing GAM applications in unrelated environmental domains. Although GAMs have been used across diverse water and environmental contexts—including coastal water quality, hydrological modelling, lake systems, ecological studies, atmospheric quality, and temporal nutrient trends (Richards et al., 2013; Murphy, Hirsch & Sprague, 2014; Richards et al., 2014; Haraguchi et al., 2015; Harding et al., 2016; Chebana et al., 2014; Liu, Feng & Wang, 2019; Yang & Moyer, 2020; Zhang & Zhi, 2020; Riemann et al., 2016; Yang et al., 2020; He, Mazumdar & Arena, 2006; Beck & Murphy, 2017; Murphy et al., 2019; Hwang et al., 2016; Laanaya, St-Hilaire & Gloaguen, 2017; Yang & Moyer, 2020)—their explicit use for PO_4_ prediction and load-relevant monitoring in irrigation return flows and drainage outlets remains limited (Murphy, Hirsch & Sprague, 2014). Existing studies have reported GAM applications for estimating nutrient concentrations and loads in rivers and at the catchment scale (Wang, Kuhnert & Henderson, 2011; Kuhnert et al., 2012; Kroon et al., 2012; Robson & Dourdet, 2015; Hagemann, Kim & Park, 2016; McDowell et al., 2021; Schramm, 2023), and recent work has explored the linkage between nonpoint-source fertilizer fluctuations and receiving-water responses (Murphy et al., 2022; Biagi et al., 2022; Schramm, 2023). However, to our knowledge, GAMs have not yet been applied to estimate PO_4_ loadings from irrigation drainage and return-flow outlets in agricultural catchments characterized by a high proportion of irrigated land; therefore, this study aims to fill this gap.

In recent years, multiple machine learning (ML) models have been applied to predict phosphorus and NO_3_ concentrations, including artificial neural networks (ANN), k-nearest neighbors (kNN), support vector machines (SVM), regression trees (RT), random forests (RF), and reduced error pruning trees (REPTree) (Mozejko & Gniot, 2008; Al-Mahallawi et al., 2012; Modaresi & Araghinejad, 2014; Ouedraogo, Defourny & Vanclooster, 2019; Bindra et al., 2019; Nieto et al., 2019; Li et al., 2020; Karamoutsou & Psilovikos, 2020; Bhattarai et al., 2021; Wu et al., 2024; Arulraj & Karthikeyan, 2024). Specifically, studies addressing PO_4_ as a target contaminant have applied machine learning as a decision-support tool, with ANN, RF, and SVM frequently used to model major pollutants (Yunus et al., 2015; Chou, Ho & Hoang, 2018; Ha et al., 2020; Shen et al., 2020; Bhattarai et al., 2021; Paepae, Bokoro & Kyamakya, 2022; Boratto et al., 2024). Other approaches include Gradient Boosting (GB) and Partial Least Squares (PLS) regression (Latif et al., 2022; Shen et al., 2020). In addition, research has attempted to estimate PO_4_ levels in water samples without laboratory processing, employing spectroscopic techniques (Zhu & Ma, 2020) and other analytical methods (Jarvie, Withers & Neal, 2002; Yokoyama et al., 2009; Boratto et al., 2024). However, fewer studies explicitly frame PO_4_ prediction for monitoring irrigation return flows at outlet points and for load-relevant assessment (concentration × discharge), which are central to evaluating the export from irrigated catchments.

The model developed in this study is intended to provide irrigation managers with valuable foresight to optimize fertilizer application, minimize PO_4_ runoff, and protect water quality. Implementing this GAM-based prediction framework can support proactive monitoring of PO_4_ at return-flow and drainage outlets, thereby improving the efficiency of monitoring programs. In practice, the model is positioned as a screening and decision-support tool that reduces monitoring time and cost by lowering the frequency of laboratory PO_4_ assays (reagent and consumable costs and instrument operating time), while preserving its ability to rapidly flag elevated concentrations and to support PO_4_ load estimation when combined with discharge data.

From a management perspective, L4 provides an integrated indicator of catchment-scale PO_4_ export (concentration × discharge) in the irrigation–drainage network. It can be used for operational screening rather than for direct hydraulic control. Drainage discharge downstream of L4 is partly reused for irrigation, and the remainder flows toward lagoon/wetland areas. Ultimately, timely detection of elevated PO_4_ at L4 in the Mediterranean Sea of Türkiye supports risk-based decisions, such as targeted sampling, evaluation of fertilizer and irrigation/drainage practices, and prioritization of mitigation actions during high-export periods.

In this context, the present study introduces a GAM-based prediction method designed to address the identified challenges. The central aim was to construct, using monitoring data from an intensively irrigated catchment, a model capable of capturing complex, nonlinear interactions between PO_4_ concentrations and multiple influential variables, including waterborne pollutants, rainfall, and hydrological flow. To justify model selection, four models—GAM, linear regression (LR), ANN, and SVM—were implemented and compared using identical training/testing splits and consistent error-based metrics; agreement between observed and predicted values was also assessed, and R^2^ was reported only as explained variance (goodness-of-fit). The proposed approach provides accurate and reliable estimates of PO_4_ concentrations, which are difficult and costly to measure, monitor, and analyze at high frequency, and offers insights into the main factors affecting this critical water-quality parameter.

## Materials & Methods

### Study area, water sampling, and analysis

The research area, the Akarsu Irrigation District (AID), covers 9,495 hectares of the Lower Seyhan Plain (LSP) in Türkiye (Alsenjar et al., 2023a; Alsenjar et al., 2023b; Karahan et al., 2024). The region is characterized by a flat, homogeneous topography and experiences hot, arid summers and mild, humid winters typical of a Mediterranean climate. According to AID records, the mean annual air temperature is 18.9 °C, with a range from 9.0 °C to 31.0 °C. Average annual precipitation in the river basin and surrounding areas is reported as 649  mm (Cetin et al., 2020). In Adana, characterized by extensive, fertile agricultural land and favorable climatic conditions, multiple crops can be cultivated in a single agricultural year. The region is known for the production of cereals, fruits, vegetables, and citrus, all of which exhibit high production efficiency (Kafalı Yılmaz, 2019; Koç & Can, 2023). In the winter of 2022, the major crops cultivated in the study area were wheat, citrus, onion, and potato. In Türkiye, summer generally lasts from June 1 to August 31, while winter extends from December 1 to February 28. Prolonged irrigation practices in this semi-arid region have affected both drainage water quality and the shallow groundwater table (Cetin et al., 2020). Excess soil water is discharged through open drainage canals, and the local soils are predominantly clay-rich. The soil composition in Akarsu comprises eleven identified soil series: Arikli, Arpaci, Incirlik, Innapli, Ismailiye, Yenice, Canakci, Mursel, Golyaka, Misis, and Gemisure (Dinç et al., 1991; Karnez et al., 2017). Among these, Arikli (29.5%), Incirlik (25.3%), and Yenice (12.2%) together account for 67% of the area. Mursel (0.7%) and Innapli (1.03%) are the least widespread (Karnez et al., 2017). Although the Lower Seyhan Plain is not generally classified as a karst region, localized karst features may occur due to underlying limestone substrata. Within the study site, groundwater levels, averaging around 1.5 m, often coincide with the crop root zone, especially during periods of rainfall and intensive irrigation. Overall, groundwater depths in the area fluctuate seasonally between 1.5 m and 3 m, with notable impacts on water table dynamics and drainage. As irrigation activities decrease toward the end of the hydrological cycle, groundwater begins to drop below the root zone. During post-irrigation periods and in the absence of precipitation, groundwater depths may reach approximately 2.5 m. Therefore, ongoing water-quality monitoring in this region is essential.

**Figure 1:**
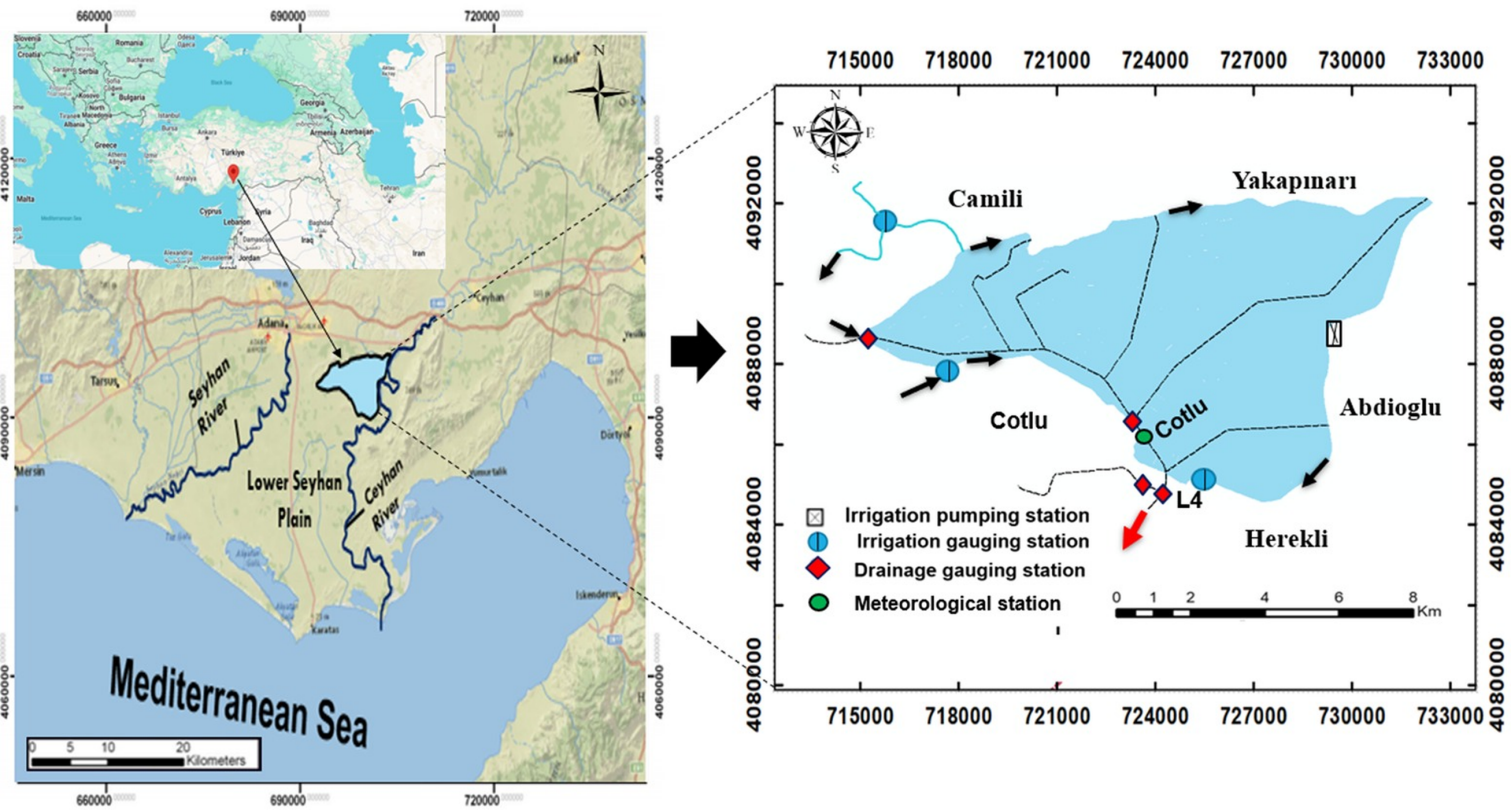
The research area’s location in Türkiye, the water sampling station (also known as the drainage gauging station), and the irrigation and drainage water flow directions.

The Akarsu Irrigation District (AID), located in Türkiye’s eastern Mediterranean region, is depicted in Fig. 1 (Karahan & Erkan Can, 2025). The figure offers a detailed representation of the Lower Seyhan Plain, highlighting the layout of its drainage and irrigation networks. It includes both a broader regional overview and an inset map providing a focused view of the study area. Directional arrows indicate the flow of irrigation and drainage within the plain.

These arrows represent the observed water flow directions based on prior hydrological surveys and operational layouts conducted in the same study area. Based on field studies and previous research in the region, the L4 location was identified as the main drainage outlet, and daily water samples were collected there. The L4 point has a strategic location for monitoring the quality and dynamics of drainage water across the Akarsu Irrigation District. The number of samples obtained from this drainage point (L4) was influenced by several factors, including field accessibility, weather conditions, and various limitations encountered during the monitoring period.

Daily water samples at the drainage gauge (L4) were collected using an automatic sampler (ISCO-3700, Louisville, Kentucky, USA) installed at the observation site. These samples were processed at the Laboratory of Agricultural Structures and Irrigation, Faculty of Agriculture, Çukurova University. Upon arrival, samples were logged, filtered through blue-band filter paper, and transferred into plastic bottles that had been cleaned with a chromic acid solution. Each bottle was accurately labeled with a unique identification number, collection date, and location to ensure traceability and prevent any misidentification during analysis. Depending on the available time and personnel, samples were either analyzed immediately (Rice & Bridgewater, 2012) or refrigerated at 4 °C until analysis. Concentrations of phosphate (PO_4_^3^^−^), nitrite (NO_2_^−^), and nitrate (NO_3_^−^) in water samples were measured in mg L^−^^1^ using Shimadzu spectrophotometers in accordance with standard methods: SM 4500-P E/Ascorbic Acid Method for phosphate as referenced in Standard Methods (2017), SM 4500-NO_2_-B for nitrite (Standard Methods, 2017), and SM 4500- NO_3_^−^-B for nitrate, (Rice & Bridgewater, 2012). Additionally, electrical conductivity (EC, dS m^−^^1^) and pH were measured using an EC meter and a pH meter in the same laboratory. Concentrations of phosphate (PO_4_^3^^−^), nitrite (NO_2_^−^), and nitrate (NO_3_^−^) in water samples were measured in mg L^−^^1^ using Shimadzu spectrophotometers in accordance with standard methods. For consistency throughout the manuscript and due to limitations in figure and table formatting, these are denoted in their simplified forms (PO_4_, NO_2_, NO_3_).

Precipitation samples were collected during rainfall events using a rainwater collection apparatus installed on the roof of the laboratory building. The system consisted of sterilized collection containers pre-positioned at an elevated location to minimize contamination from surrounding surfaces or airborne particles. The apparatus was securely fastened and remained in place throughout the rainfall event, ensuring that only direct atmospheric precipitation was captured under controlled, consistent conditions.

Based on routine laboratory practice and our experience during the analytical phase of this study, the preparation and analysis time per sample differed among the parameters. Nitrite (NO_2_^−^) and nitrate (NO_3_^−^) analyses required relatively short preparation times, typically on the order of 10–20 min per sample, as these methods involve fewer reagents and simpler preparation steps. In contrast, phosphate (PO_4_^3^^−^) analysis using the ascorbic acid method required a longer preparation and reaction time, generally 20–40 min per sample, due to additional reagent preparation, color development, and stabilization steps.

In terms of analytical cost and material consumption, phosphate analysis was more resource-intensive than nitrite and nitrate. The PO_4_^3^^−^ method requires a larger number of reagents, including mixed color reagents with limited shelf life, resulting in higher per-parameter consumable costs. Conversely, NO_2_^−^ and NO_3_^−^ analyses rely on fewer reagents and simpler workflows, leading to lower material consumption and lower cost per parameter.

Therefore, among the analyzed parameters, phosphate (PO_4_^3^^−^) analysis consumed more laboratory time, reagents, and overall cost per sample compared to nitrite and nitrate, while NO_2_^−^ and NO_3_^−^ analyses were faster and more economical.

### Observed data used

The dataset used in the modeling process was derived from laboratory analyses of collected water samples and from flow data recorded at the drainage monitoring station. Daily laboratory measurements of PO_4_, NO_2_, and NO_3_ constituted the core of the data. Concurrent values for NO_3_, Q (discharge), EC, and P (precipitation) were obtained from the dataset presented by Karahan & Erkan Can (2025). The dataset comprised 522 daily observations from the 2022 and 2023 water years in the Lower Seyhan Plain. It was structured as a 522  × 7 matrix and it included six input variables (EC, pH, Q, P, NO_2_, NO_3_) and observed PO_4_ concentrations as the response variable. This complete dataset was used for model development and evaluation.

As previously stated, because direct PO_4_ measurement is difficult and costly, this study proposes a method that estimates PO_4_ values from a limited set of alternative variables that are more practical to monitor, even when datasets are incomplete or irregular. In this context, correlations between PO_4_ levels and NO_2_, NO_3_, EC, pH, Q, and P were computed, as detailed in the Results section. Because the dataset is a daily time series and exhibits short-lag persistence, we used a time-structured (blocked/contiguous) split to avoid information leakage between training and testing and to evaluate the model’s generalization capability. This time-structured split is the basis for the main performance metrics reported; random split repetitions are presented only to demonstrate robustness to the choice of partition. Specifically, the dataset was first ordered by date, and the first 80% of observations (earlier period) were used for model training, while the final 20% (later period) were reserved for testing. This preserves the 80/20 ratio while ensuring that the test set chronologically follows the training set. Random 80/20 splits, repeated 100 times, were retained solely for a secondary sensitivity analysis and were not interpreted as unbiased estimates of forecast skill under autocorrelation.

### Benchmark models for comparison (LR/ANN/SVM)

To benchmark the proposed GAM framework and assess whether commonly used alternative approaches can predict PO_4_ in irrigation return-flow and drainage water at the L4 outlet, three additional predictive models were implemented: LR as a conventional baseline model, and two widely used machine-learning approaches, artificial neural networks (ANN) and support vector machines (SVM), both of which have been applied in water-quality and nutrient prediction studies (Helsel et al., 2020; Yunus et al., 2015; Chou, Ho & Hoang, 2018; Ha et al., 2020; Shen et al., 2020). All benchmark models were trained using the same input variables (EC, pH, Q, P, NO_3_, NO_2_) and the same observed PO_4_ dataset to ensure comparability. Because the dataset is a daily time series and exhibits short-lag persistence, the benchmark used the same time-structured (blocked/contiguous) 80%/20% split as the GAM did to avoid information leakage between training and testing. Specifically, the data were ordered by date; the first 80% of observations (earlier period) were used for training, and the final 20% (later period) were reserved for testing. Unless otherwise stated, the reported LR/ANN/SVM results correspond to this time-structured split. Predicted PO_4_ concentrations were compared with observed values at the L4 outlet using a consistent evaluation framework based primarily on error metrics (RMSE, MSE, and bias). The coefficient of determination (R^2^) was reported only as explained variance (goodness of fit). Overall model performance was assessed by examining how closely predicted values aligned with observations relative to the 1:1 relationship. To assess sensitivity to partition choice, random 80%/20% splits were repeated across 100 independent trials; however, these random-split results are reported only as a secondary sensitivity analysis and are not interpreted as unbiased estimates of forecast skill under autocorrelation.

### Generalized additive model and application

This research used a GAM to estimate phosphate (PO_4_) concentrations in irrigated environments. As noted earlier, GAMs are a flexible category of statistical models that effectively capture non-linear relationships between predictors and the response variable (Huang et al., 2020; Wang et al., 2023). The model was trained using the comprehensive dataset and applied to predict PO_4_ levels. Because observations are collected daily and may exhibit short-term autocorrelation, temporal structure was incorporated by adding a smooth term for time (date/time index) and allowing an AR(1) correlation structure in the residuals using a GAMM framework. Accordingly, the GAMM specification and residual ACF diagnostics are used to address temporal dependence rather than assuming independent observations. Temperature was not included as an explicit predictor because the objective is a minimal-input, outlet-screening model; instead, broad seasonal/thermal and crop-cycle effects are represented by a smooth function of time (date/time index). After model fitting, residual temporal dependence was assessed as part of the diagnostic workflow; therefore, the model was specified with a temporal smoother and an AR(1) residual structure (GAMM) to explicitly account for autocorrelation rather than assuming independence. To limit model complexity, the GAM was fitted using penalized smooth functions, so that model flexibility was controlled by smoothness penalties rather than by freely increasing the degrees of freedom. Model generalization was evaluated using a time-structured (blocked/contiguous) 80%/20% split, with the earlier period used for training and the later period for testing, thereby reducing information leakage from autocorrelation. Because the aim of this study is outlet-scale prediction, model comparison and interpretation focus on out-of-sample error metrics under time-structured validation, rather than on AIC-based selection among many alternative GAM specifications. Model predictive performance was primarily assessed using error-based metrics (MSE, RMSE, bias/mean error, and error SD), while R^2^ was reported only as explained variance (goodness-of-fit). The findings affirm the ability of the proposed approach to predict PO_4_ concentrations effectively, making it a valuable tool for managing the quality of drainage and irrigation water. The GAM methodology is a powerful tool for statistical modeling and regression analysis. GAM is commonly expressed in the following formulation:

\begin{eqnarray*}Y={\beta }_{0}+{f}_{1}(X\mathrm{_}1)+{f}_{2}({X}_{2})+\cdots +fp(X\rho )+\epsilon \end{eqnarray*}

Y: Dependent variable (response variable)

*β*o: Constant term (intercept)

f_1_, f_2_,…,f_p_: Non-linear functions for the independent variables (X_1_, X_2_, …,X_p_). These functions are typically referred to as smooth functions and are used to model nonlinear relationships in the data.

€: Error term

GAM uses spline- and kernel-based techniques to model nonlinear relationships. These functions allow for more flexible modeling of independent variables (Wood, 2006; Hastie & Tibshirani, 1990a). Therefore, GAM can learn complex, nonlinear relationships in the data more effectively than other regression models. In the general formulation of a GAM, a separate function is defined for each independent variable f_j(X_j).

While GAM offers substantial flexibility for modeling complex non-linear relationships, this flexibility must be managed carefully to avoid overfitting. Excessive use of smoothing functions (*e.g.*, too many splines) can reduce the model’s predictive performance on new data, underscoring the importance of balanced model specification.

## Results

### Pre-modeling data analysis and model implementation

As emphasized, this study aims to develop a method for estimating PO_4_ levels using readily accessible, reliably measurable parameters, particularly when data may be incomplete or irregular due to the high cost and complexity of direct PO_4_ analysis. To this end, the daily distributions of the input variables and PO_4_ were first examined.

**Figure 2:**
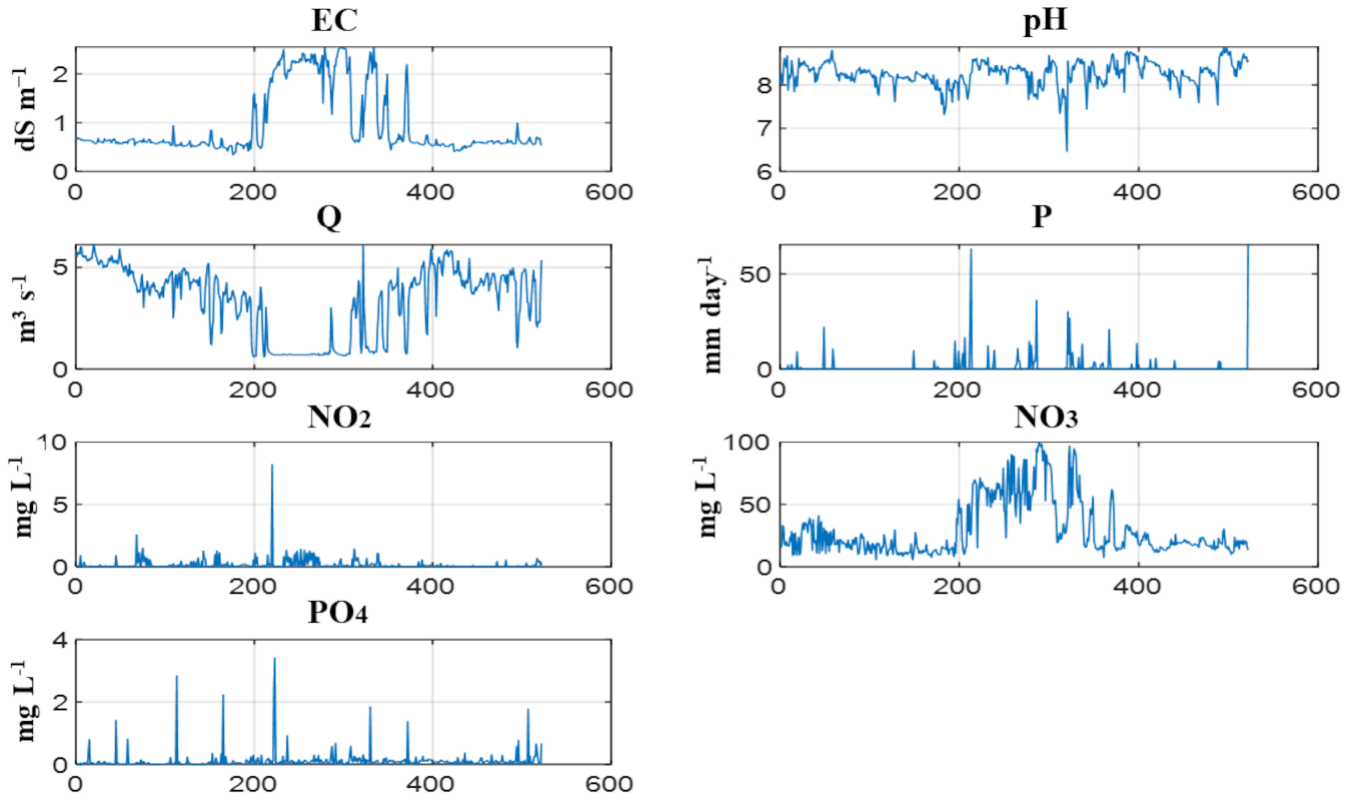
Daily time series of model input variables and measured PO_4_ (*X*-axis shows sampling date). The observation period, 2022-04-27 to 2023-09-30.

Figure 2 presents the temporal distribution of the input variables and PO_4_ utilized in the GAM model. The plotted time series reveal considerable variation across parameters, reflecting both natural hydrological dynamics and external anthropogenic influences over the monitoring period.

The maximum EC value was measured in January 2023. The pH values remain relatively stable, fluctuating within a narrow, slightly alkaline range typical of surface irrigation waters in agricultural areas. Discharge (Q) and precipitation (P) exhibited peaks consistent with rainfall events and seasonal flow regulation, with sharp fluctuations suggesting short-term hydrological responses. Nutrient-related variables—including NO_3_, NO_2_, precipitation (P), and PO_4_—exhibit more irregular temporal patterns. In particular, NO_3_ concentrations increased markedly between November 2022 and March 2023, with values occasionally exceeding 80 mg L^−^^1^. This pattern is likely associated with seasonal fertilizer applications and drainage from intensively cultivated areas. PO_4_ concentrations remained generally low but exhibited sporadic spikes, indicating potential point-source contamination or runoff-induced surges during or following precipitation events. Because downstream impact is more directly related to phosphorus export (load) than to concentration alone, variations in concentration should be interpreted in conjunction with discharge (Q). In this study, PO_4_ concentration is modeled as an outlet-scale screening signal; when the modeled PO_4_ is combined with the routinely measured Q at L4 (PO_4_ × Q), the resulting predictions can support the identification of periods with potentially elevated phosphorus export. The non-linear and intermittent behavior of several variables supports the selection of a GAM approach, as it allows flexible fitting of nonparametric trends and interaction structures among predictors over time. In line with the study objective of developing an outlet-scale PO_4_ screening model using a minimal set of routinely monitored inputs, the predictors were limited to EC, pH, Q, P, NO_2_, and NO_3_. The seasonal and phenological structure apparent in Fig. 2 (regime shifts and seasonal blocks) was explicitly represented in the GAMM by a smooth function of time (date/time index), which serves as an operational proxy for broad seasonal drivers—including temperature-related effects on concentrations and crop-cycle timing—without adding monitoring requirements. Although incorporating measured temperature could further strengthen process-based interpretation, the purpose here is to provide a parsimonious and transferable model suitable for settings where only routine outlet variables are consistently available. Accordingly, the interpretation focuses on how routinely measured outlet conditions, together with time-varying seasonal forcing, explain observed PO_4_ variability in irrigation return-flow and drainage water.

The observed temporal variability in the input parameters (Fig. 2) highlights the complexity and heterogeneity of the water quality dynamics in the study area. Such variability, particularly nutrient and agricultural surges and nonlinear hydrological patterns, underscores the need for a modeling framework capable of capturing intricate temporal dependencies. Accordingly, the GAM was implemented to predict PO_4_ concentrations using this temporally rich dataset. The following sections detail the model structure, variable selection process, and evaluation metrics and assess model performance and the relative importance of predictor variables. To interpret predictor contributions in the fitted GAM (beyond overall R^2^), we examined variable-wise partial effects using Partial Dependence Plots (PDP) and Accumulated Local Effects (ALE). These diagnostics show the direction and shape of each predictor’s influence on predicted PO_4_ and highlight that the effects are nonlinear and concentrated within specific value ranges. The main “critical ranges” and effect directions inferred from the ALE/PDP curves are summarized in the tables to support transparent interpretation of the model’s behavior rather than treating it as a black box.

**Figure 3:**
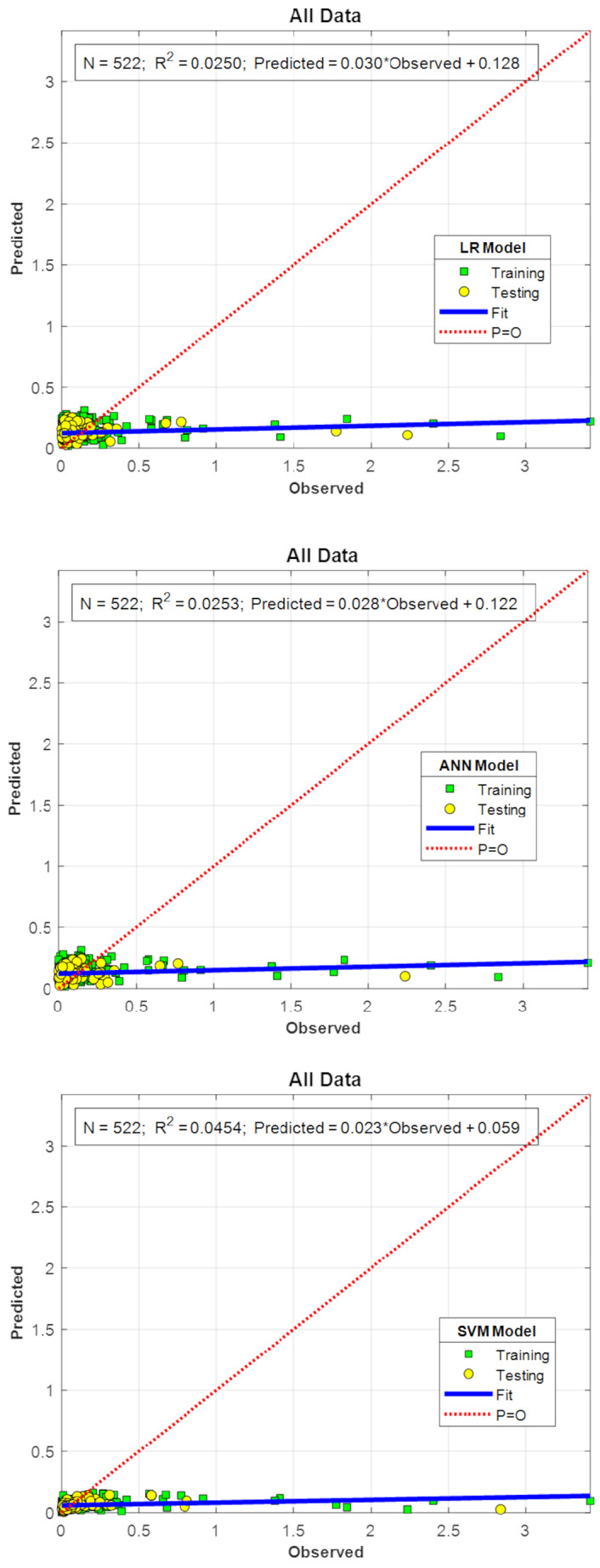
Predicted *versus* observed PO_4_ concentrations for benchmark models Linear Regression (LR), Artificial Neural Network (ANN), and Support Vector Machine (SVM). Training and testing points are shown in each panel. The red dotted line (P = O) denotes the 1:1 reference line (Predicted = Observed), indicating perfect agreement. The blue solid line (“Fit”) is the least-squares line fitted to predicted *versus* observed values to summarize systematic bias (slope and intercept) relative to the 1:1 line.

The results indicate that PO_4_ exhibits low-to-moderate correlations with classical water quality parameters (EC, pH, Q, P, NO_2_, and NO_3_). Although the strongest relationship was detected with NO_3_, the overall correlation coefficients were weak. Therefore, flexible modeling techniques were explored to improve predictive performance. In addition to the GAM approach, commonly used machine learning models, including LR, ANN, and SVM, were evaluated for comparison. This comparison is presented in Fig. 3.

Figure 3 illustrates the predictive performance of LR, ANN, and SVM using predicted-versus-observed PO_4_ scatter plots. For each method, the model was trained on the training subset and then applied to the held-out test subset; predictions for both the training and test subsets are displayed in the same panel for transparency. Despite hyperparameter tuning, predictive accuracy remained low, with *R*^2^ = 0.0250 for LR, 0.0253 for ANN, and 0.0454 for SVM. The visual clustering (“compression”) of points near low observed values mainly reflects the PO_4_ distribution, which contains many low concentrations and relatively few high spikes; therefore, a linear-scale plot becomes dense near the origin. Under a weak linear or monotonic signal, LR/ANN/SVM tend to produce near-constant predictions, which manifest as a nearly horizontal fitted (“Fit”) line (*i.e.,* a least-squares line fitted to Predicted *versus* Observed values) and as large departures from the 1:1 (Predicted = Observed) reference (“P=O”) line during high PO_4_ events. In contrast, the GAM, which models nonlinear smooth effects, captured the multivariate nonlinear structure more effectively and achieved substantially better agreement with observations (testing *R*^2^ = 0.7875). This highlights the robustness and suitability of the GAM approach for capturing phosphate dynamics under heterogeneous environmental conditions, outperforming conventional linear and non-linear alternatives.

**Figure 4:**
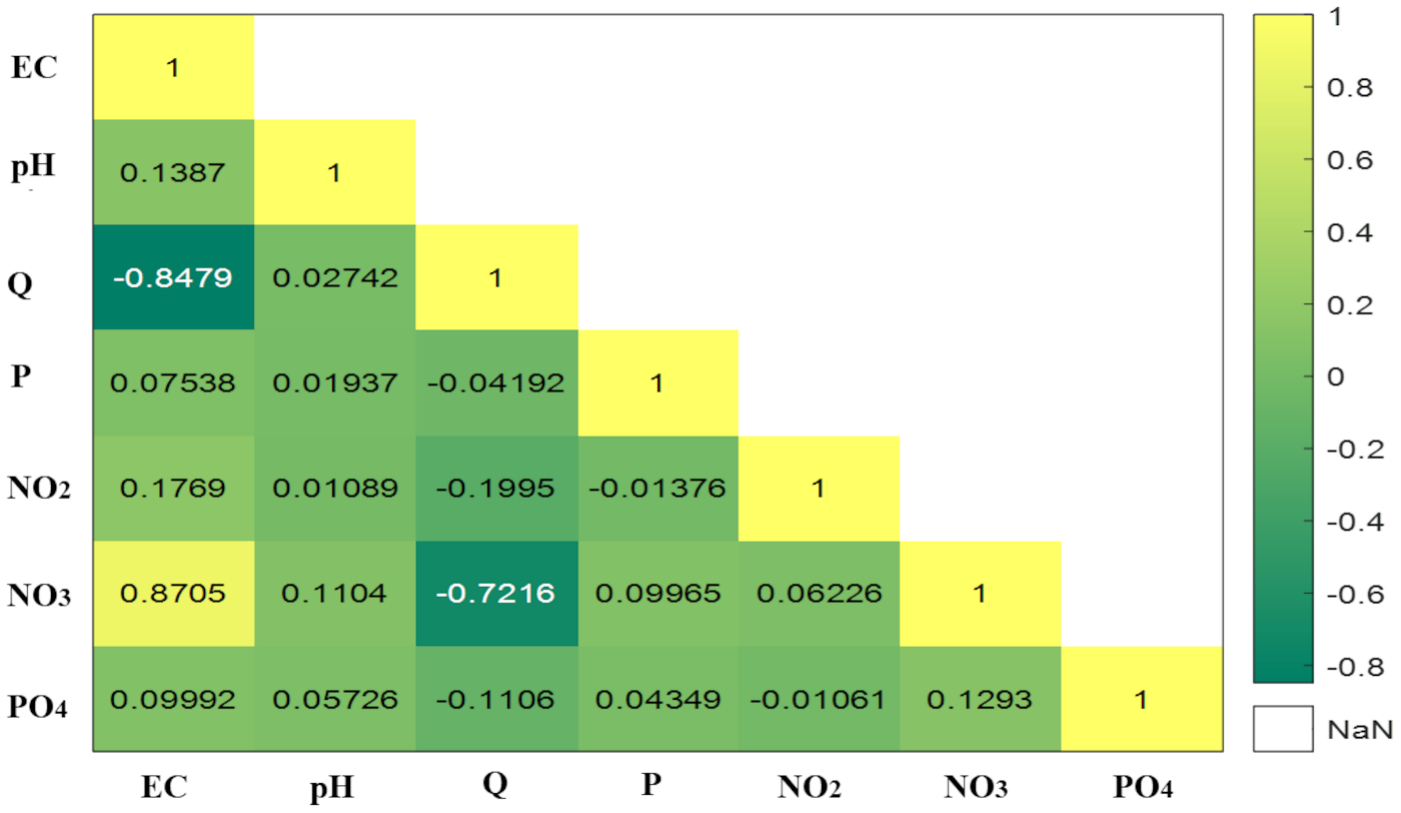
Lag-0 (same-day) Pearson correlation matrix between PO_4_ and model parameters (reported as descriptive summaries for this autocorrelated daily time series).

Figure 4 shows exploratory Pearson correlations at lag = 0 (same-day associations). Because the variables are daily time series with autocorrelation, these coefficients are used only for descriptive summaries, and not for statistical inference or predictor selection. Potential delayed responses are instead accounted for within the modeling and evaluation framework by using a time smoother and an AR(1) term in the GAMM, and by applying time-structured validation.

As shown in the correlation matrix in Fig. 4, a very strong positive relationship (*r* = 0.87) was observed between EC and NO_3_. This indicates that electrical conductivity increases with the ionic content of the water. In contrast, a strong negative correlation (*r* =  − 0.85) was observed between EC and flow rate (Q). The decrease in EC values under high-flow conditions indicates a dilution effect. Similarly, the strong negative correlation (*r* =  − 0.72) between NO_3_ and Q was associated with a decrease in nitrate concentrations under high-flow conditions.

Overall, the lag-0 correlations between PO_4_ and the routine predictors are small, indicating that strong same-day linear associations are limited in this dataset. This does not imply the absence of relationships because PO_4_ responses can be nonlinear, conditional, and lagged with respect to hydrological forcing. Accordingly, conclusions are drawn from the time-structured GAMM results and out-of-sample error metrics, rather than from Pearson r values alone.

**Figure 5:**
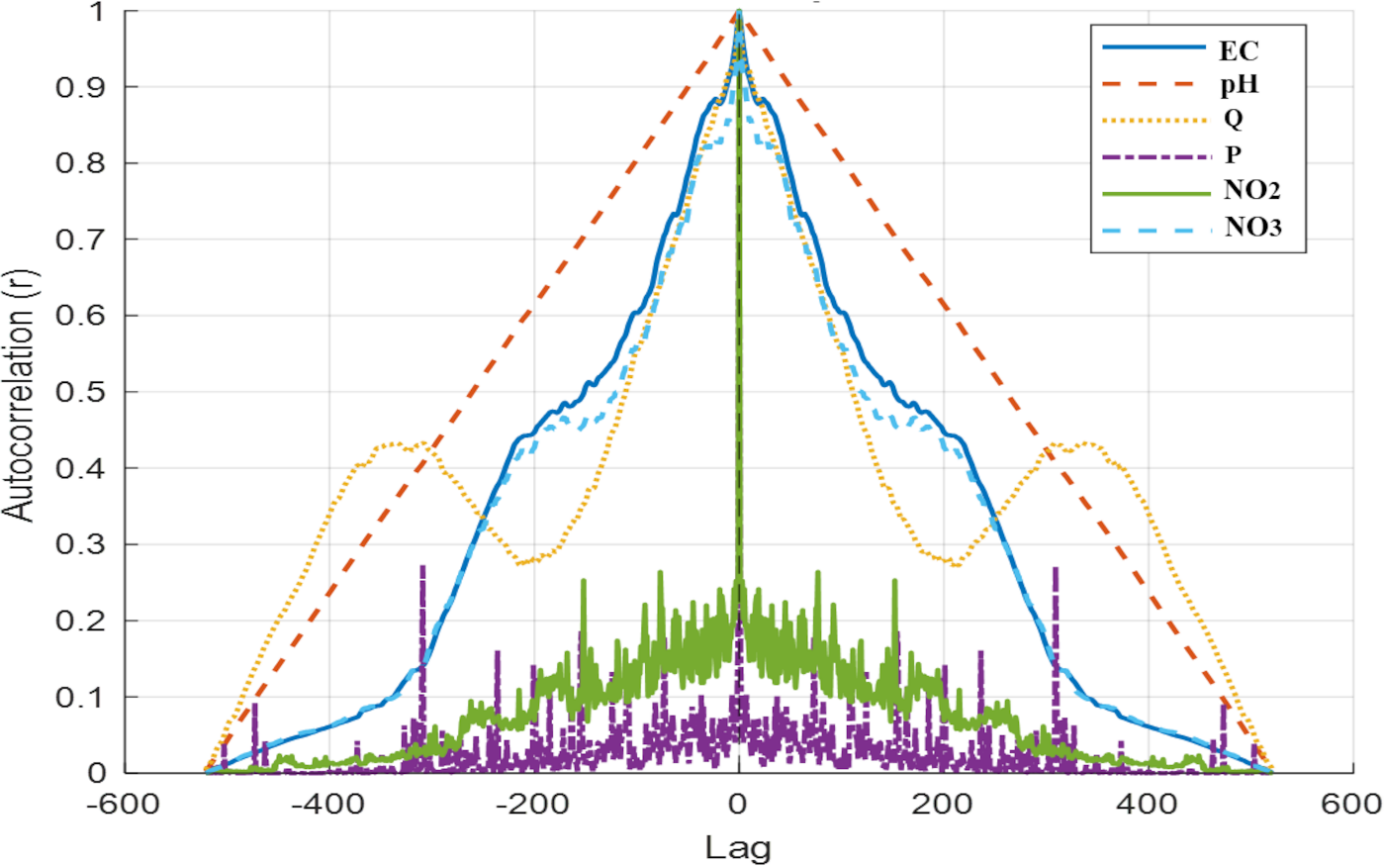
Autocorrelation functions (ACFs) of the input variables (EC, pH, Q, P, NO_2_, NO_3_). Dashed horizontal lines indicate the 95% confidence bounds (±1.96/√ N) around zero; autocorrelation values outside these limits are considered statistically significant.

In the autocorrelation analysis, the maximum coefficient (*r* = 1 at lag = 0) was found for all variables (Fig. 5), which is expected, as it reflects the identity of a series with itself. Several variables also showed short-lag persistence, indicating that daily observations are not fully independent even though no strong periodicity or clear cyclic repetition was evident across forward or backward time shifts. To support the interpretation of the ACF, Fig. 5 was revised to include 95% confidence bounds (±1.96/√N),enabling visual identification of statistically significant autocorrelation at nonzero lags. Accordingly, the subsequent GAM specification accounts for the temporal structure of the data by including a smooth function of time and allowing for autocorrelated residuals *via* an AR(1) correlation structure during model fitting. This combination reduces the risk of overstating predictor significance and improves uncertainty estimation under temporally dependent errors. Lagged versions of selected predictors (*e.g.*, 1–3-day lags) were evaluated as candidate features and retained only when they improved out-of-sample error metrics (RMSE and MSE) under time-structured validation to avoid unnecessary model complexity. Autocorrelation in predictor time series was not used as a selection criterion; instead, predictors (EC, pH, Q, P, NO_2_, NO_3_) were selected based on routine availability, mechanistic relevance to outlet-scale PO_4_ dynamics, and their contribution to out-of-sample performance under time-structured validation. Because the present study focuses on daily outlet-scale prediction, lag selection and AR(1) handling are treated as sensitivity components; the final specification is reported, including the chosen time-smoother basis dimension and the estimated AR(1) parameter (*ρ*).

**Figure 6:**
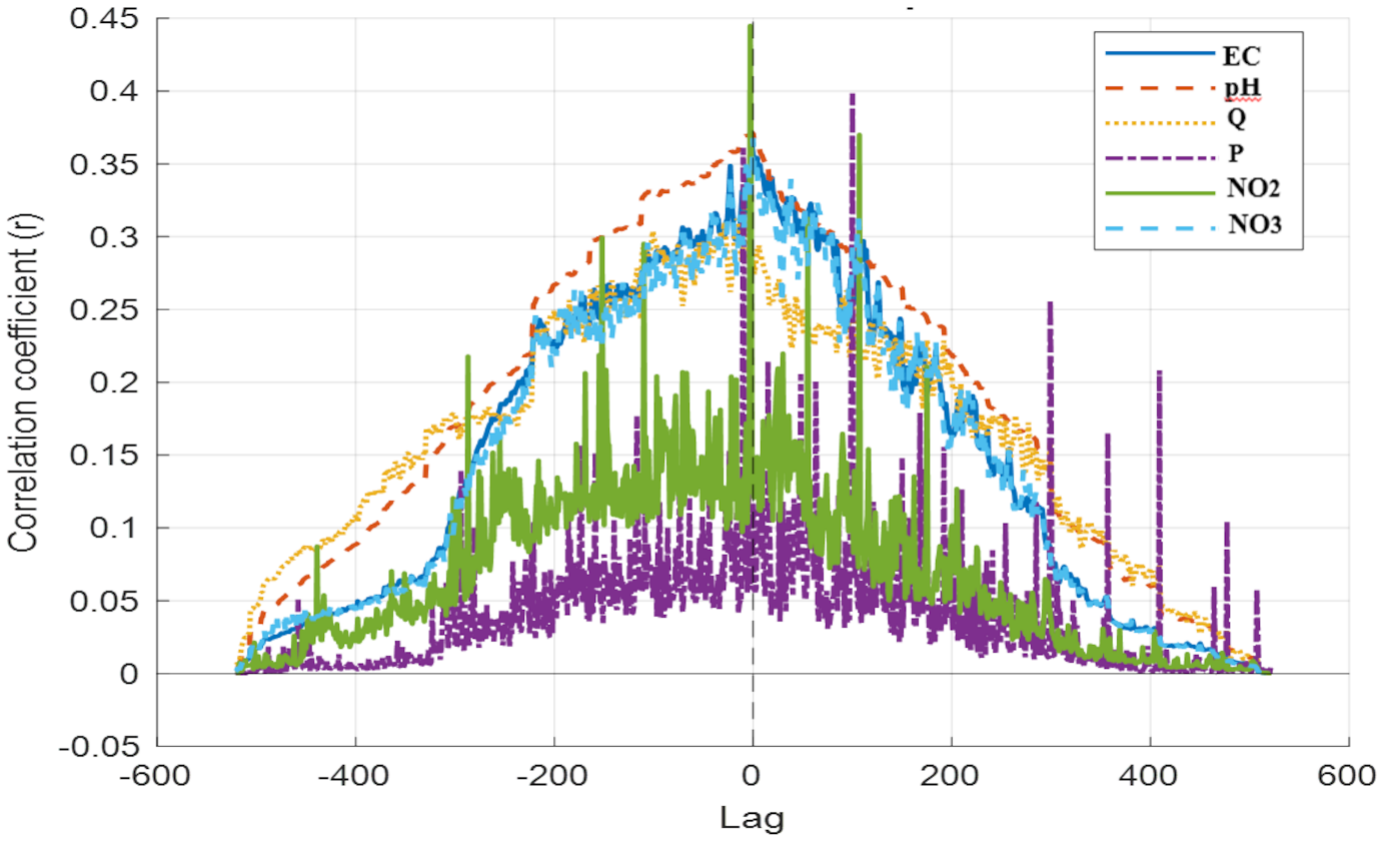
Exploratory cross-correlation functions (CCF) between PO_4_ and predictor variables across time lags. CCF values were computed on standardized series with lag-dependent overlap; therefore, lag-0 CCF values are not expected to be numerically identical to the lag-0 Pearson correlations in Fig. 4. The CCF is used to screen plausible lag directions (candidate lags) rather than for inference.

Because lagged relationships are plausible in hydrochemical time series, an exploratory cross-correlation function (CCF) analysis is also reported (Fig. 6). Note that the CCF differs from the Pearson correlation matrix in Fig. 4 because it uses standardized (mean-removed) series and lag-dependent overlap; therefore, lag-0 values are not interpreted as direct equivalents of Pearson r.

To examine the relationships between PO_4_ and input variables in more detail, a cross-correlation analysis was performed because lagged relationships are plausible in hydrochemical time series; the cross-correlation results (Fig. 6) are reported to summarize delayed associations in an exploratory manner. As shown in Fig. 6, the highest correlations were observed for NO_2_, P, EC, pH, and NO_3_. These patterns were used solely to identify candidate short lags (*e.g.*, 1–3 days) for sensitivity testing; lag terms were retained only when they improved out-of-sample error under time-structured validation.

CCF results are reported as exploratory screening for plausible lag directions and are not used as inferential evidence for causal strength. When cross-correlations between phosphate and input variables were examined, the strongest correlation was observed with NO_2_ (*r* = 0.44, lag = −3). This finding suggests that changes in PO_4_ may be related to NO_2_ concentrations several time steps earlier.

A moderate correlation (*r* = 0.40) was detected between P and PO_4_, but the fact that this relationship emerged at lag = +100 suggests it may be linked to long-term rather than short-term processes. Additionally, weak to moderate relationships were found between PO_4_ and pH (*r* = 0.37, lag = –1), between PO_4_ and NO_3_ (*r* = 0.37, lag = 0), and between PO_4_ and EC (*r* = 0.36, lag = –1). The relationship between Q and PO_4_ (*r* = 0.32; lag = −8) was weaker.

Among these, the strongest positive correlation with PO_4_ was observed for NO_3_ (*r* = 0.1293), suggesting that NO_3_ and PO_4_ may share common sources, such as agricultural fertilization practices. Notably, the relationship between PO_4_ and flow rate is negative. PO_4_ exhibited a weak negative correlation (*r* =  − 0.1106) with flow rate (Q). This pattern may reflect a dilution effect, in which higher water volumes in the drainage channels dilute PO_4_ concentrations despite continued inputs from agricultural areas. Although this correlation is weak, it demonstrates that GAM captured subtle, non-linear interactions and predicted PO_4_ levels with good accuracy in both training and testing. Similarly, weak positive correlations were observed between PO_4_ and EC (*r* = 0.09992) and between PO_4_ and pH (*r* = 0.05726), suggesting a minimal direct influence under the studied conditions. The complex nonlinear structure of environmental systems, comprising numerous interdependent components, is reflected in the low correlation values between PO_4_ and other input variables. As shown in Fig. 4, although the correlation between PO_4_ and other parameters was low, GAM learned this relationship during the proposed model’s training phase and subsequently predicted the test data quite accurately.

PO_4_ concentration is an important indicator of water quality in aquatic systems. The GAM model was implemented using the 522 × 7 dataset, with six water quality parameters as predictors and PO_4_ as the target variable. The following sections present details of the implementation of the GAM model for predicting PO_4_ fluctuations in the Lower Seyhan Plain. For model evaluation, the dataset was split using a time-structured (blocked/contiguous) 80/20 split (earlier period for training, later period for testing) to reduce information leakage from autocorrelation. Repeated random 80/20 splits were used only for sensitivity analysis.

The GAM model achieved strong predictive performance, with R^2^ values of 0.8319 on the training dataset and 0.7875 on the test dataset. Its robustness was further supported by 100 independent trials using randomly selected subsets, confirming the model’s capacity to generalize beyond a single data configuration. The average R^2^ across these 100 runs was 0.772, with a standard deviation of 0.0166, indicating stable and reliable results. This finding underscores the model’s effectiveness and adaptability across varied data structures without compromising prediction accuracy.

Table 1 presents the statistical description of the parameters used in the GAM-based PO_4_ prediction model. As shown in Table 1, PO_4_ concentrations ranged from 0.00 to 3.42 mg L^−1^, with a mean of 0.12 mg L^−1^ and a standard deviation of 0.29 mg L^−1^. This range indicates pronounced temporal variability in PO_4_ levels at the L4 drainage outlet, reflecting fluctuations across the irrigation system over time rather than spatial variability. While concentration statistics describe variability, management relevance is often expressed as export (load). Because Q is measured at the outlet and included in the dataset, the predicted PO_4_ time series can be directly translated into a load-oriented screening indicator (PO_4_ × Q) when needed. Likewise, other input variables, such as EC, pH, discharge (Q), precipitation (P), NO_2_, and NO_3_, exhibited wide variations. For example, NO_3_ concentrations ranged from 0.02 to 50.85 mg L^−1^, whereas EC ranged from 0.66 to 2.38 dS m^−1^. The relatively high standard deviations observed across several parameters highlight the dataset’s heterogeneity. This situation illustrates the challenge of fitting a model to the given data. This level of variability is beneficial for modeling, as it highlights the model’s ability to generalize and capture complex, nonlinear interactions among variables. Therefore, the dataset’s diversity contributed to the robustness and predictive performance of the implemented GAM.

**Table 1:** Statistical description of the models’ input parameters and PO_4_.

	**EC** **(dS m** ^−1^ **)**	**pH**	**Q** **(m** ^**3**^ ** s** ^−1^ **)**	**P** **(mm)**	**NO**_**2**_ **(mg L** ^−1^ **)**	**NO_3_** **(mg L** ^−1^ **)**	**PO_4_** **(mg L** ^−1^ **)**
**Min**	0.34	6.47	0.59	0.00	0.00	5.53	0.00
**Max**	2.55	8.88	6.12	65.40	8.21	99.57	3.42
**Aveg**	0.95	8.27	3.37	1.16	0.19	29.66	0.12
**StdD**	0.66	0.28	1.70	5.36	0.46	22.21	0.29

**Figure 7:**
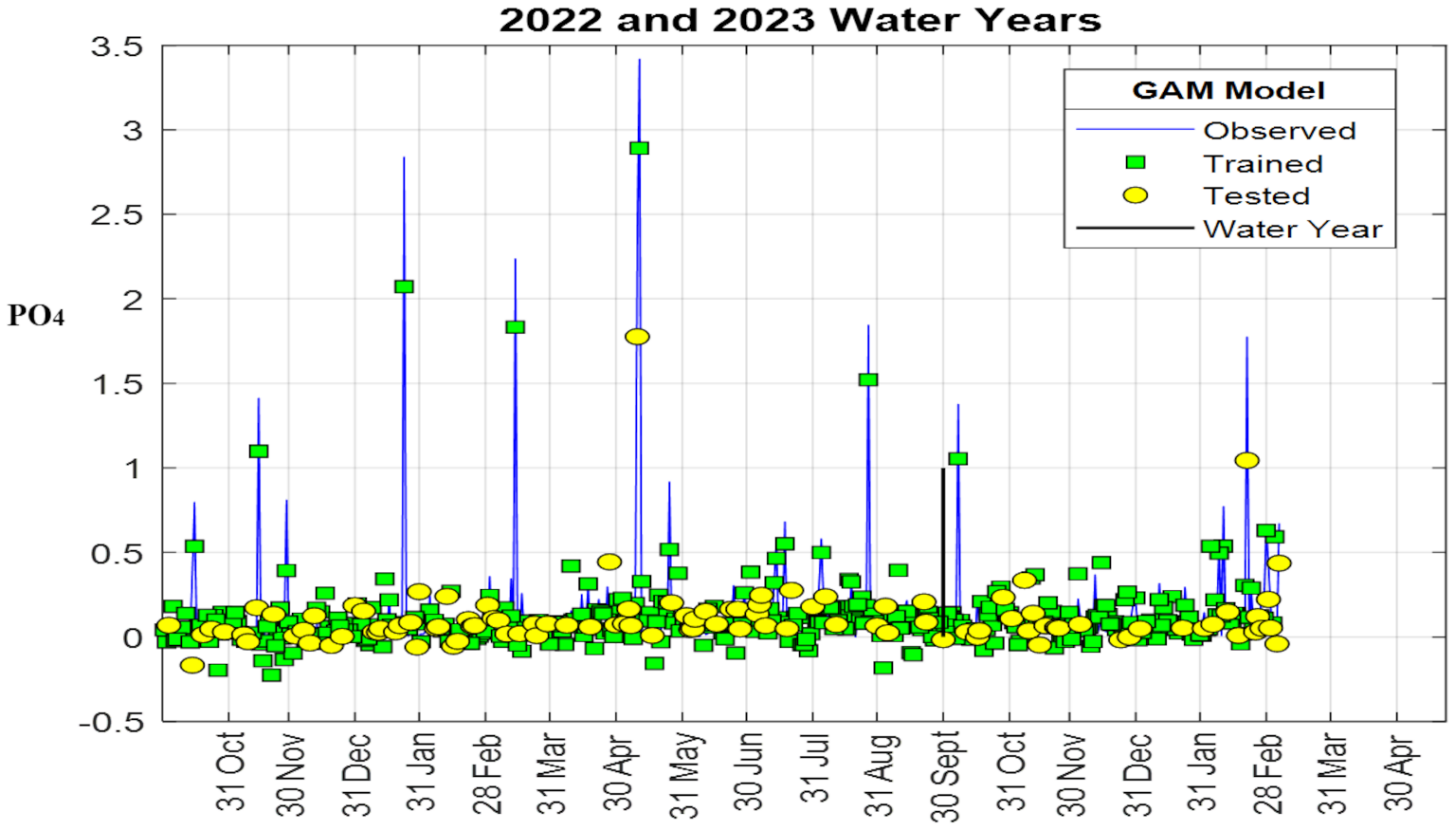
Variation of observed PO_4_ (continuous line) and GAM predictions through time.

Figure 7 illustrates the model’s training and test results, compared with the observed data (continuous line). Because the plot overlays observed values with both training and test predictions, it is visually dense and is intended as a qualitative overview of agreement over time rather than a detailed diagnostic of temporal errors. The model captures the general temporal dynamics in both the training and testing phases, though it underestimates some high peak concentrations. The temporal structure of errors is more clearly assessed using the residual time series and the error histogram.

Green squares and yellow circles indicate predictions for the training and test subsets, respectively. Because all points are shown together, the figure provides an overview of temporal agreement.

**Figure 8:**
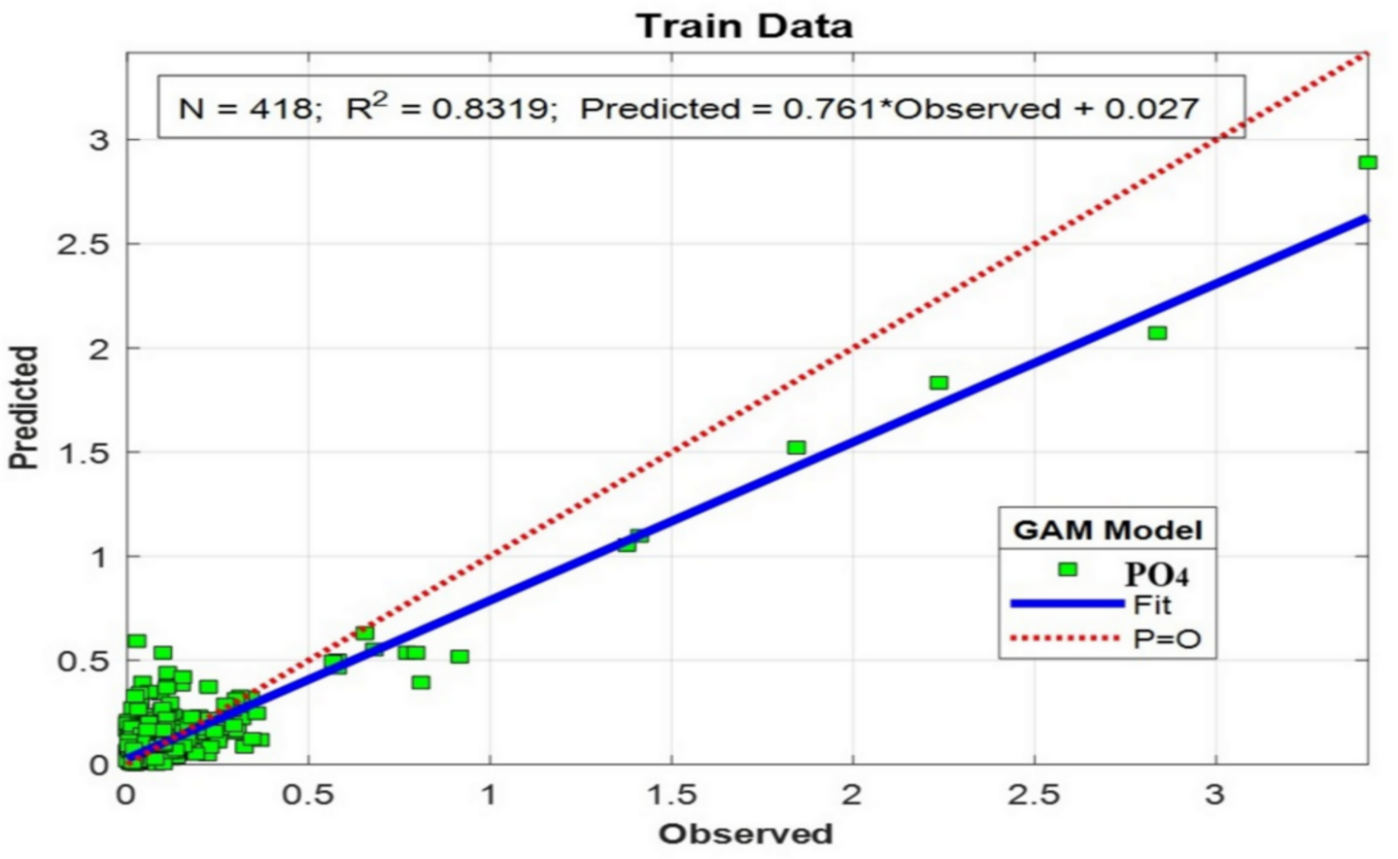
Scatter plot of PO_4_ values used in training the model.

**Figure 9:**
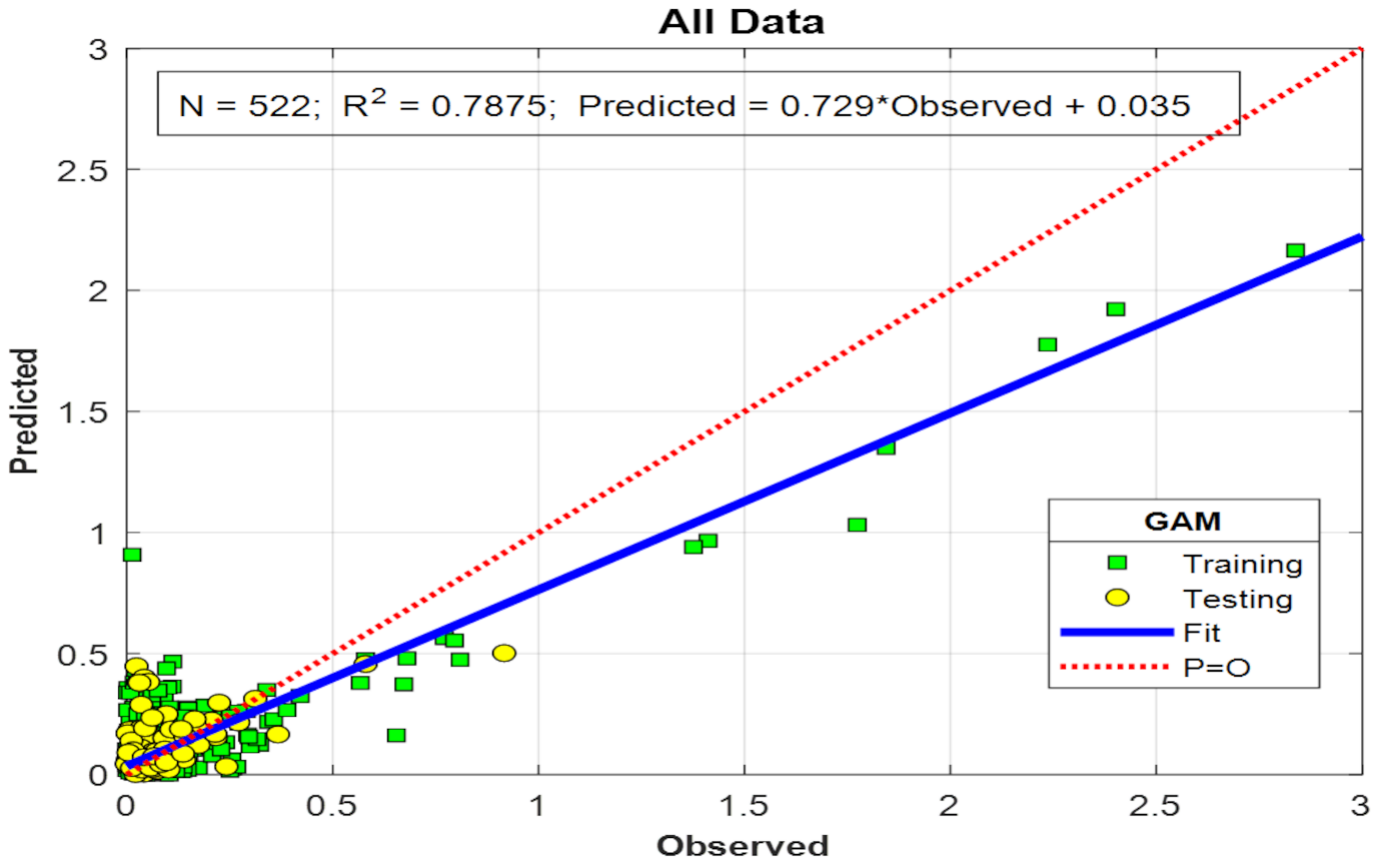
Scatter plot of PO_4_ values used in training and testing the model.

Figure 8 shows the scatter plot of the data used for model training and the PO_4_ values predicted by the model. As shown in Fig. 7, the model effectively learned the large values in the 418 data points used in the training phase. In Fig. 9, an R^2^ of 0.7875 was obtained between observed and modeled results across 522 PO_4_ data points. The R^2^ value obtained during model training (0.8319) indicates that the GAM has high predictive performance.

To assess model performance, we examined the distribution of residuals (observed minus predicted values). The residuals were approximately normally distributed and centered near zero for most observations, indicating minimal bias and an adequate representation of overall variability. This is consistent with prior studies showing that GAMs are well suited to water-quality modeling where nonlinear relationships and interactions are important (Hastie & Tibshirani, 1990b; Wood, 2017).

The clustering of low PO_4_ values observed in Figs. 8 and 9 can be attributed to both the dataset’s statistical properties and the environmental context of the study area. As indicated in Table 1, the mean PO_4_ concentration is 0.12 mg L^−^^1^, while the standard deviation is 0.29 mg L^−^^1^—more than twice the mean. This suggests a highly skewed distribution, with most data concentrated near the lower values and a small number of high values, creating a long tail. Such unevenness explains the clustering of points at low PO_4_ values in the scatter plots and reflects the system’s natural PO_4_ imbalance.

Environmentally, this clustering is further supported by variability in fertilizer application routines and by differences in the rates at which pollutants originating from these fertilizers reach water resources. Factors such as soil type, irrigation scheduling, crop uptake efficiency, and rainfall intensity affect phosphorus mobility and runoff potential. Consequently, the observed clustering is not a model artifact but rather an accurate representation of the realistic framework of PO_4_ dynamics in the studied system, which the GAM successfully captures and adapts to.

The model captured temporal fluctuations well despite low correlations. Additionally, the model predicted PO_4_ values that deviated from the observed values in some periods. The distributions of the PO_4_ values in the training data and in the model outputs (Fig. 8) clearly show that the model predicts larger PO_4_ values more accurately. It demonstrates strong agreement between predicted and observed values during the training phase. It also revealed that the model’s predictive ability was strong as concentrations increased. As shown in the figure summarizing the model’s performance on the training and test sets (Fig. 9), the R^2^ value was 0.8319 for the training set and 0.7875 for the test set. These values indicate that the model generalizes well and performs well on the test data. The small difference between the test and training R^2^ values demonstrates the model’s strong generalization ability. The points gathered along the diagonal line in the graph visually confirm the model’s successful predictive performance. The histogram of the model’s prediction errors indicates that the distribution is approximately normal. That the error distribution is close to the zero-error line strongly indicates high overall model performance. The apparent contradiction between weak individual linear correlations (maximum *r* = 0.1293) and strong model performance (*R*^2^ = 0.832) can be attributed to the GAM’s ability to capture complex, nonlinear, and interactive relationships that traditional correlation analysis may not detect. While Pearson correlation coefficients measure only linear associations between variables, PO_4_ dynamics in agricultural systems are governed by multiple interacting factors, including non-linear threshold effects, interactive effects, temporal lag effects, and conditional relationships. Critical ranges and localized nonlinear effects of key predictors were identified using ALE and PDP analyses (Table 2), confirming the GAM’s ability to capture subtle dynamics that are not evident from linear correlations.

### Correlation structure supported by ALE and PDP analyses

**Figure 10:**
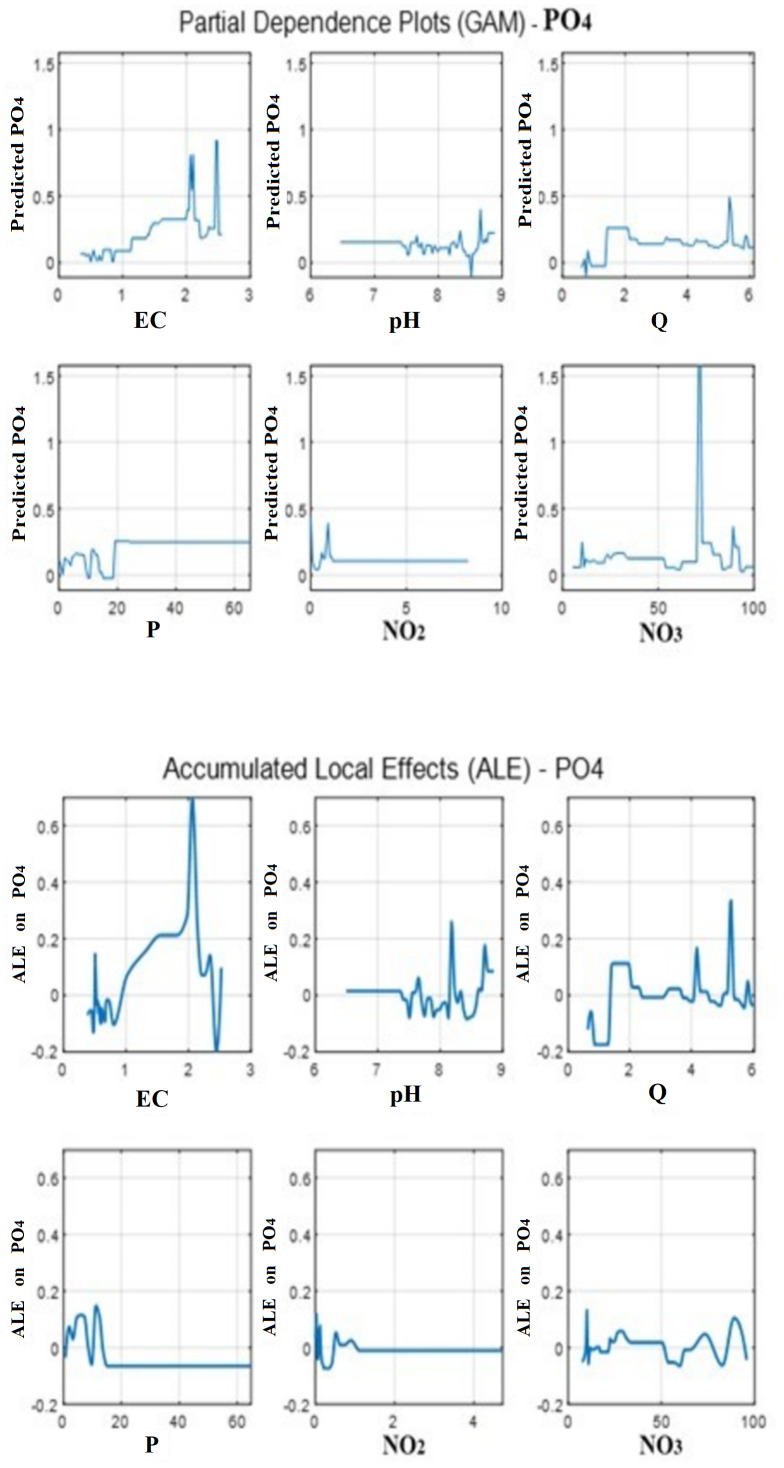
Partial Dependence Plots (top) and Accumulated Local Effects (bottom) for EC, pH, Q, P, NO_2_, and NO_3_ in the fitted GAM, showing the nonlinear, range-specific contributions of each predictor to PO_4_ predictions.

Because Pearson correlations capture only linear same-day associations, we interpret predictor contributions primarily using GAM partial-effect diagnostics (PDP and ALE). Figure 10 and Table 2 summarize the direction, nonlinearity, and predictor ranges over which PO_4_ is most sensitive. Accordingly, predictor contribution is interpreted from these GAM partial effects and the summarized critical ranges (Table 2), rather than from lag-0 Pearson r values.

An examination of the correlation matrix showed that the relationships between PO_4_ and the environmental predictors were generally weak. Positive correlations were observed for PO_4_ with EC (r ≈ 0.10) and NO_3_ (r ≈ 0.13), and these patterns are supported by the corresponding Accumulated Local Effects (ALE) and Partial Dependence Plots (PDPs) (Fig. 10). The ALE curves indicate that PO_4_ predictions increase slightly as EC and NO_3_ increase, confirming the model’s sensitivity to these predictors despite their low linear correlations.

Correlation values for pH, P (precipitation), and NO_2_ were minimal (*r* < 0.06), and their ALE/PDP curves similarly showed a negligible influence on PO_4_ predictions across most of their ranges. In contrast, Q exhibited a negative correlation with PO_4_ (r ≈ −0.11), and its ALE curve confirmed that increases in Q were associated with a slight decrease in predicted PO_4_ concentration. Collectively, these findings demonstrate that the tendencies observed in the correlation matrix are consistent with the non-linear effects learned by the GAM model, as represented in the ALE and PDP visualizations (Fig. 10).

**Table 2:** Critical ranges and effect directions of key water quality variables according to the GAM model.

**Variable**	**Critical range/Feature**	**Direction of effect**
**EC**	1.8–2.1	Increase followed by decrease (peak around 2.0)
**pH**	8.15–8.20	Increase
**Q**	5.25–5.30	Slight decrease
**P**	0–15	Variation; >15 flat
**NO_2_**	0–1	Variation; >1 flat
**NO_3_**	Across domain (−0.05 to −0.1)	Fluctuating, low

Although linear correlations were weak, the ALE plots clearly revealed that several predictors exerted non-linear and range-specific effects on PO_4_ predictions. For EC, the predicted PO_4_ showed a sharp increase for values beyond 1.8, reached a maximum around 2.0, and declined rapidly after 2.1. For pH, a distinct rise was observed within the 8.15–8.20 interval. The Q variable showed a slight decrease in predicted PO_4_ values within the range 5.25–5.30. For P, the ALE curve showed variation over the range 0–15 and became completely flat thereafter, indicating that the model used P only within this limited range. NO_2_ exhibited a clear effect between 0 and 1, but became negligible beyond this range. NO_3_ exhibited small oscillations between −0.10 and −0.05 across its domain, reflecting a weak but nonrandom effect. These observations confirm that the GAM successfully captures local nonlinear patterns that simple linear correlations cannot detect.

### Non-linear effects and critical value ranges

When the correlation matrix and ALE/PDP results were jointly evaluated (Table 2), it was evident that the GAM model extracted meaningful, localized non-linear patterns from the data. EC, pH, Q, P, and NO_2_ each exerted significant effects within optimal intervals, and had limited influence outside those ranges. NO_3_ demonstrated a low but fluctuating influence across its full range. Importantly, these results explain why linear models and other approaches—whose predictive performance was substantially lower (as confirmed by their hyperparameter analyses)—failed to capture these dynamics. In contrast, the generalized additive model (GAM) leverages smooth, data-driven non-linear functions, enabling it to learn relationships that are weak in a global linear sense but strong within localized segments. This alignment between the correlation matrix and the GAM’s nonlinear learning behavior underscores the model’s internal validity and interpretability.

**Figure 11:**
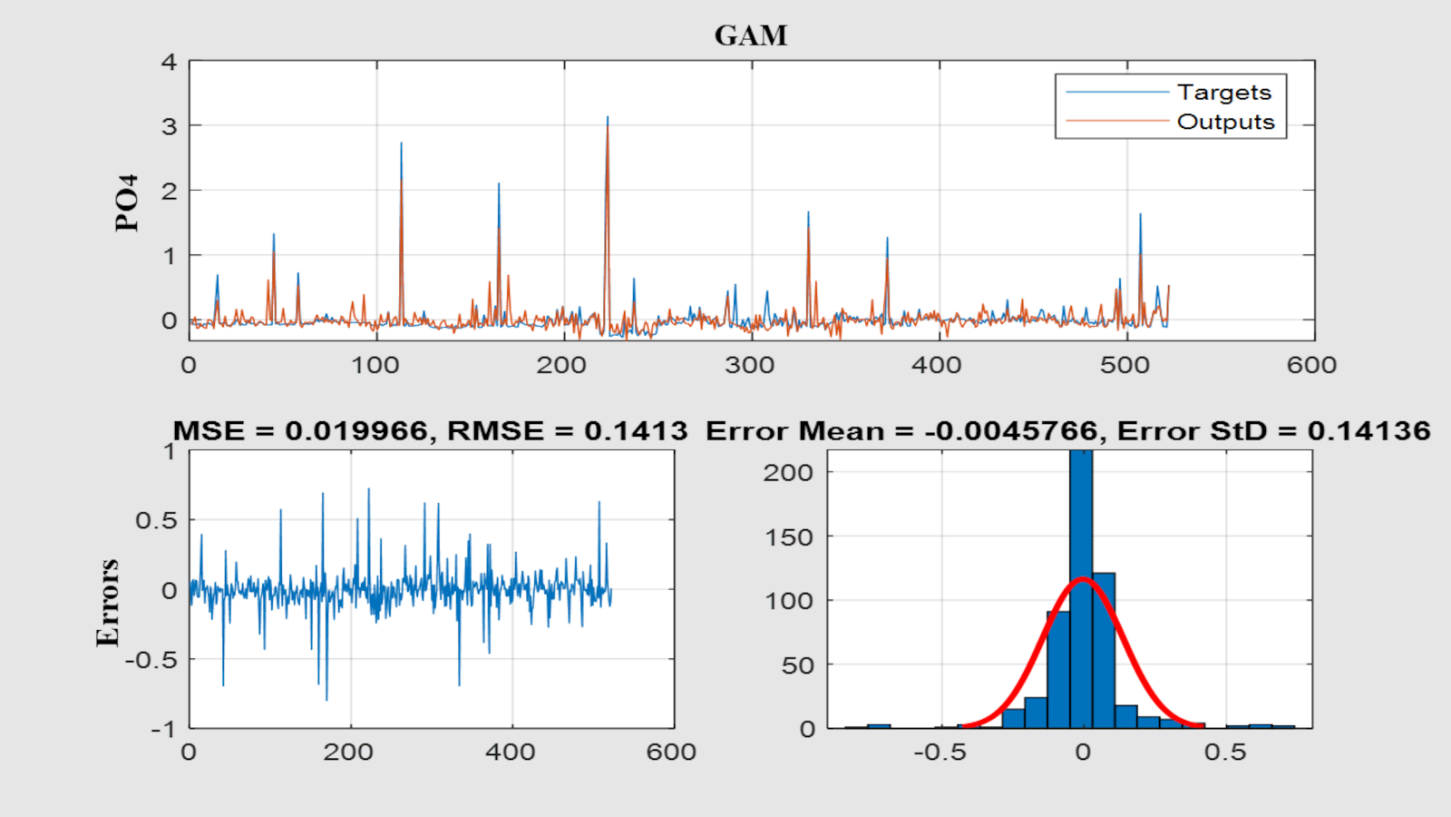
Time-series comparison of observed and predicted PO_4_ values, residual time series, and histogram of residuals.

To further evaluate model performance and the distribution of residuals, additional analyses were performed. Figure 11 presents the time-series comparison between observed and predicted PO_4_ values, the error time series, and the error histogram. These results confirm that the model effectively captures the overall temporal dynamics, while high peak concentrations are slightly underestimated. The residuals were centered around zero and largely followed a normal distribution, as shown in the histogram. A few extreme values deviated from this pattern, yet the general trend remained consistent with normality. Presenting the time series alongside the error diagnostics in Fig. 11 provides a comprehensive overview of the model’s performance across the full dataset.

## Discussion

### Correlations between PO_4_ and other water quality parameters

As shown in Fig. 4, the strongest positive correlation with PO_4_ was observed for NO_3_ (*r* = 0.1293), suggesting that both nutrients may originate from common agricultural sources. Similarly, in a study by Bhattarai et al. (2021), various machine learning algorithms were employed to estimate NO_3_ and P concentrations in five watersheds with different land uses, revealing positive relationships between these nutrients in agricultural areas. A notable correlation between NO_3_ and PO_4_ supports the hypothesis of shared agricultural sources, while their weak negative association with flow rate suggests potential dilution during periods of increased discharge.

Flow rate (Q) showed a weak negative correlation (*r* =  − 0.1106), suggesting a dilution effect during high-discharge events. EC (*r* = 0.09992) and pH (*r* = 0.05726) also showed weak positive correlations with PO_4_. Unlike NO_2_, NO_3_, Q, and P, EC and pH are comparatively easy to measure as water quality metrics. EC and pH are also closely linked to geochemical processes that affect PO_4_ dynamics. EC, in particular, reflects the presence of dissolved ions that directly affect water chemistry and nutrient transport, while pH plays a key role in phosphorus solubility and availability. The minimal correlations observed for EC and pH imply a limited direct influence under the study’s specific conditions. The US Environmental Protection Agency (USEPA, 1987) reports that EC is meaningfully related to total PO_4_ levels.

These findings reflect the complex and interconnected nature of nutrient transport and support the use of a nonlinear modeling approach to better capture these relationships. Collectively, these weak linear associations underscore the need for a flexible modeling framework, such as a GAM, to effectively account for nonlinear and interacting processes within the environmental system. This complexity supports the selection of the GAM, which is particularly effective at capturing such non-linear relationships. The GAM demonstrated its resilience in simulating PO_4_ dynamics by learning the underlying patterns and generating accurate predictions despite the weak linear correlations. Latif et al. (2022) investigated the performance of four machine-learning models for predicting PO_4_ concentrations in the Feitsui Reservoir.

The correlation between PO_4_ and NO_3_ was 0.3246, considered very weak; however, it was the highest among the parameters. Therefore, in that study, ammonia, magnesium, nitrate, total dissolved solids (TDS), and total hardness were used as inputs for the proposed models. Although the methods, study area, and water sample sources differed, a fundamental consistency was observed in our study.

### Dataset characteristics and modeling approach

PO_4_ concentration serves as a crucial indicator of water quality. The predictive framework was based on 522 daily records from the 2022 and 2023 water years, linking six water-quality indicators (EC, pH, Q, P, NO_2_, NO_3_) to observed PO_4_ levels. Because the dataset is a daily time series, model evaluation was based on a time-structured (blocked/contiguous) 80/20 split to reduce information leakage due to autocorrelation; repeated random 80/20 splits were used only as a sensitivity analysis. The literature states that 70–80% of the data should be used for training, with the remainder used for testing (Maier & Dandy, 2000; Karahan et al., 2014). Among several error metrics examined, mean squared error (MSE) was adopted because of its sensitivity to large deviations, which makes it a robust criterion for assessing model performance for datasets with high temporal variability (Karahan, 2012; Yagiz, Yazitova & Karahan, 2020; Karahan, 2014).

The results show that the GAM method has the highest accuracy in predicting PO_4_ concentration. Temporal patterns of the variables revealed nonlinear behavior and episodic fluctuations. EC exhibited a seasonal increase; pH remained stable; precipitation and discharge showed episodic peaks; nutrient variables, particularly NO_3_ and PO_4_, displayed irregular fluctuations with occasional surges. These irregularities support the selection of GAM, which can capture such complex temporal dynamics.

### Phosphorus transport, soil, and environmental context

A significant proportion of total phosphorus (TP) delivered to rivers originates from surface runoff from watersheds, predominantly in particulate form (Persson, 2001; Sharpley et al., 2013), and is strongly associated with suspended solids (Sandström et al., 2020). Theoretically, this suggests a positive correlation between TP concentrations and river discharge. However, even with extensive datasets, TP–discharge relationships are often weak under baseflow conditions. This may be attributed to the complex nature of watershed hydrology (McDonnell et al., 2007), including attenuation processes that reduce sediment-associated TP transport in larger catchments (Novotny, 1980; Roehl, 1962), nutrient cycling, land use impacts, anthropogenic contributions, and inherent data uncertainties (Harmel & Smith, 2007; Almeida & Coelho, 2023). Predicting total phosphorus (TP) and other phosphorus fractions, for example particulate phosphorus (PP) or dissolved reactive phosphorus (DRP), becomes much more challenging with small datasets owing to two main difficulties: (i) a limited ability to include additional predictors; and (ii) difficulty identifying the drivers of forecast variability. Regardless of their features, the models will not perform well if the predictors do not capture the sources of variability in predictions (Almeida & Coelho, 2023). Comparison of Fig. 2 with the literature indicates that the mentioned working conditions correspond to the complex structure described therein. The dataset was relatively small and exhibited a heterogeneous distribution. In addition, although there is partial consistency among other parameters in the dataset, this consistency is not reflected in the PO_4_ data.

In addition to these factors, soil properties in the basins are of great importance. According to Jenny (1994), soil-forming factors such as climate, time, parent material, terrain, and vegetation all influence the regional variability of soil parameters. These findings indicate that the predominantly clay-rich soils in the study area are likely to influence both the heterogeneity of the observed data and model performance. However, it is mainly influenced by anthropogenic activities (Jenny, Salem & Wallis, 1968; Yigini & Panagos, 2016; Rodrigo-Comino et al., 2018). Under the influence of human factors, soil productivity in dry and semi-arid regions can vary greatly over short distances. This situation can be explained by multifactorial effects in basins with intensive agricultural production (Kaya et al., 2022).

As reported in Table 1, the dataset exhibits pronounced variability across both nutrient concentrations and physicochemical parameters. Such heterogeneity reflects the dynamic nature of the irrigation environment and underscores the importance of adopting modeling approaches that can accommodate nonlinear, nonstationary relationships. Rather than being adequately explained by central tendencies alone, these data highlight fluctuations and extreme values that must be captured to support effective management.

A dissolved PO_4_-P concentration of approximately 0.02 mg L^−^^1^ in aquatic environments is considered sufficient to trigger algal proliferation (USEPA, 1995). The World Health Organization (WHO, 2011) and the European Union (1998) established the maximum allowable nitrate concentration in drinking water at 50 mg L^−^^1^, whereas the USEPA (2007) recommended a slightly lower threshold of 45 mg L^−^^1^. The WHO (2011) also indicates a permissible limit of 3 mg L^−^^1^ for NO_2_ from agricultural sources, and the WHO (2011) sets acceptable surface water limits of 50 mg L^−^^1^ for NO_3_ and 5 mg L^−^^1^ for PO_4_.

The descriptive statistics presented in Table 1 reflect the water quality characteristics of the study area. PO_4_ levels ranged from 0.00 to 3.42 mg L^−^^1^, with an average of 0.12 mg L^−^^1^. When assessed against internationally recognized water quality standards, these concentrations frequently exceeded the ecological threshold of 0.02 mg L^−^^1^ reported by USEPA (1995), indicating a potential risk of eutrophication within the irrigation drainage system. Similarly, NO_3_ concentrations—though generally within the limits set by WHO (2011), the European Union (1998), and the USEPA (2007)—occasionally surpassed these thresholds, raising concerns about potential health risks if the water is consumed without treatment. NO_2_ concentrations also exceeded the WHO (2011) guideline value of 3 mg L^−^^1^ in some samples, highlighting acute toxicity risks and potential adverse impacts on both human health and aquatic ecosystems.

### Methodological considerations

An important methodological limitation of this study is the inability to remove seasonality from the dataset. Although seasonal effects were initially examined, the data lacked sufficient temporal coverage for a reliable seasonal decomposition. The dataset spans 522 days; some months occur twice, while others appear only once, resulting in an uneven temporal distribution. Such limited, imbalanced coverage prevents the identification of statistically meaningful seasonal cycles and reduces the robustness of attempts to estimate seasonal components. Seasonality was therefore not removed, and all analyses were conducted using the dataset’s raw temporal structure. In addition, because predictor–PO_4_ relationships can change with management practices or hydrological regime, model performance should be periodically checked (*e.g.*, tracking RMSE and bias) and the model recalibrated if skill declines. Thus, the approach can reduce the frequency of PO_4_ assays, but periodic confirmatory sampling remains necessary for long-term deployment. Collectively, these findings underscore the system’s environmental vulnerability and emphasize the need for robust predictive tools to monitor nutrient dynamics effectively. In particular, elevated nutrient levels in some samples raise significant concerns about risks to water quality. In conclusion, the dataset demonstrates instances where nutrient concentrations—especially PO_4_ and NO_2_—exceed ecologically and health-relevant thresholds. These results highlight the importance of regular monitoring and implementing mitigation strategies to safeguard environmental and public health, especially when drainage waters reach natural water bodies or when cumulative effects are considered. In agricultural drainage waters, total phosphorus (TP) concentrations have been reported between 15% and 50%, with the corresponding dissolved PO_4_-P concentration ranging from 0.05 to 1 mg L^−^^1^ (Uslu & Türkman, 1987; Coşkun, 1995). These findings highlight the variability in phosphorus speciation across different nutrient-loading conditions in agricultural runoff. Bilgiç & Bahçeci (2014) investigated the transport of NO_3_, P, potassium (K), pH, total dissolved solids (TDS), and sediment load in drainage systems of the Harran Plain. Water samples were collected from 10 open drainage channels and 15 subsurface drainage outlets at 15-day intervals during the irrigation season. Results indicated that phosphorus and potassium were present only at trace levels, whereas the NO_3_ load varied significantly, ranging from 31 to 69 mg L^−^^1^. These findings highlight the dominant dynamics of nitrogen pollution in the region’s agricultural drainage waters. Peker, Azgın & Celik (2018) assessed the impact of agricultural activities on the quality of drainage water in the Develi Plain. The authors reported 24-month average values for EC, pH, and nitrate-nitrogen (NO_3_-N): 586 µmhos cm^−^^1^, 7.9, and 0.5 mg L^−^^1^, respectively. Regarding PO_4_-P, the maximum, minimum, and average concentrations were found to be 0.30 mg L^−^^1^, 0.00 mg L^−^^1^, and 0.07 mg L^−^^1^, respectively. Iskenderoglu (2009) examined seasonal variation in orthophosphate (o-PO_4_) concentrations in the TD8 and TD0 drainage canals within the Right Bank Irrigation Area of the Lower Seyhan Plain. The study reported a wide range of o-PO_4_ values across five monitoring stations during 2007–2008. At Station 1, o-PO_4_ concentrations ranged from 0.20 mg L^−^^1^ to 5.8 mg L^−^^1^; at Station 2, the measured value was 0.57 mg L^−^^1^ in June 2008; at Station 3, concentrations ranged from 0.21 mg L^−^^1^ to 5.35 mg L^−^^1^; at Station 4, o-PO_4_ levels ranged from 1.2 mg L^−^^1^ to 5.00 mg L^−^^1^; and at Station 5, the values fluctuated between 0.22 mg L^−^^1^ and 8.4 mg L^−^^1^. The findings highlight substantial spatial and temporal variability in o-PO_4_ concentrations within the drainage network. Notably at Station 5, elevated o-PO_4_ levels were attributed to reverse inflows from the Mediterranean Sea, indicating a secondary nutrient-loading source in addition to local agricultural runoff. This suggests a heightened risk of eutrophication in downstream water bodies, driven by both anthropogenic and hydrodynamic influences. The results were largely consistent with those of previous similar studies. It was concluded that the observed differences, including higher values, were due to variations in location and impact among the sampled drainage channels.

Recent studies have increasingly applied machine learning techniques to model and predict water quality parameters in various aquatic systems. For example, Latif et al. (2022) assessed the predictive capabilities of four machine learning models—ANN, SVM, RF, and Boosted Trees (BT)—for estimating PO_4_ levels in the Feitsui Reservoir. They used monthly water-quality records from 1986 to 2014 to develop the model. The models incorporated five input variables: ammonia, magnesium, nitrate, total dissolved solids (TDS), and total hardness. Among the tested models, ANN exhibited the highest predictive accuracy, achieving a Root Mean Squared Error (RMSE) of 1.199, a Mean Absolute Error (MAE) of 0.858, a Mean Squared Error (MSE) of 1.439, and an R^2^ of 0.979. The researchers concluded that the ANN model provides a robust tool for managing eutrophication in water systems. Similarly Bhattarai et al. (2021) evaluated nine machine learning algorithms for predicting concentrations of NO_3_ and phosphorus across five watersheds with varying land uses. The study found that the ML models provided more accurate predictions of P concentrations across all watershed types. Among the models tested, the Ensemble-BO algorithm demonstrated the best performance, with the coefficient of determination (R^2^) ranging from 0.709 to 0.878. Although the methods, sampling points, and input parameters differ across the literature, the usability of artificial intelligence applications for estimating PO_4_ was consistent with the study’s results.

Differences between this research and earlier studies can be attributed primarily to (i) sampling frequency and temporal differences (our study uses daily sampling, *n* = 522 samples, which better captures short-duration spikes), (ii) sampling location (the main drainage outlet, L4, rather than multiple localized channels), and (iii) hydrodynamic and secondary-source influences. These methodological and hydrodynamic distinctions explain why values in our daily dataset can differ from those reported in studies with lower sampling frequency or different station coverage. The findings of this study are consistent with the literature, showing high temporal and spatial heterogeneity in phosphorus concentrations in agricultural drainage systems. The use of daily data, combined with a GAM-based modelling framework, enhances the detection and prediction of short-term PO_4_ surges that less frequent monitoring may miss. This strengthens the novelty and practical relevance of our study for the management of PO_4_ in drainage waters. The daily sampling approach (*n* = 522) captures short-duration PO_4_ spikes missed by conventional periodic sampling. While Peker, Azgın & Celik (2018) reported PO_4_-P ranges of 0.00–0.30 mg L^−^^1^ using periodic sampling, the daily monitoring revealed a range of 0.00–3.42 mg L^−^^1^, which better represents the true variability in agricultural drainage systems. This spatial integration also includes cumulative effects and dilution processes that are not apparent in localized sampling. By sampling at the main drainage outlet (L4), this study captures integrated drainage for the entire 9,495-hectare district, unlike point-specific measurements from individual channels in previous studies. Moreover, this study addresses a gap in the literature by applying GAMs to the prediction of PO_4_ in agricultural drainage systems, despite weak linear correlations (maximum *r* = 0.1293). This approach aligns with Latif et al. (2022), who achieved successful predictions using related but different methods despite weak correlations (*r* = 0.3246 for PO_4_-NO_3_).

While machine learning techniques are widely applied in groundwater quality assessments (Schilling et al., 2020), their use in estimating phosphorus levels in groundwater is comparatively limited. Model effectiveness is influenced by various factors, including model type, dataset size, precision, and the nature and quantity of input variables (Bui et al., 2020). Prediction of phosphorus in groundwater requires adaptable modeling algorithms and the minimization of variables and data requirements to enhance model practicality. Streamlining variable selection is crucial to reducing model complexity and improving usability. The factors impacting shallow groundwater phosphorus concentrations are multifaceted, encompassing surface phosphorus input loads, soil characteristics, infiltration mechanisms, and groundwater physicochemical parameters (Yang et al., 2023). Despite factors and limitations noted in the literature, an analysis of variation in measured PO_4_ levels and GAM-predicted PO_4_ levels over time (Fig. 2) shows that the model captures peak values accurately and follows a trend parallel to the observed data.

## Conclusions

Monitoring of PO_4_ at irrigation return-flow and drainage outlets is important because these outlets integrate catchment-scale nutrient mobilization and indicate when exported phosphorus may increase downstream eutrophication risk and compromise reuse of drainage water.

This study aimed to develop a parsimonious, outlet-scale screening model to estimate daily PO_4_ concentrations in irrigation return-flow and drainage water at L4 from routinely monitored variables, thereby reducing reliance on frequent laboratory PO_4_ assays. The key novelty lies in the use of high-frequency (daily) drainage outlet observations (*n* = 522, 2022–2023 water years) together with a minimal set of routinely available predictors (EC, pH, Q, P, NO_2_, NO_3_) in an interpretable GAM framework tailored for operational deployment at drainage outlets.

Using 522 daily observations from the Lower Seyhan Plain (Adana, Türkiye), the GAM framework reproduced observed PO_4_ variability well, despite weak pairwise linear correlations, demonstrating that outlet PO_4_ dynamics are governed by nonlinear, multivariate relationships rather than by strong same-day linear associations. Predictive performance demonstrated strong agreement between measured and estimated PO_4_ (training *R*^2^ = 0.8319, testing *R*^2^ = 0.7875) and low overall bias (mean error near zero), with error magnitudes summarized by RMSE and MSE. Model performance is primarily evaluated using error-based metrics (RMSE, MSE, and bias), whereas R^2^ is reported as the explained variance (goodness-of-fit). Because the dataset is a daily time series, temporal dependence was explicitly addressed by including a smooth function of time and fitting an AR(1) residual structure, and model skill was assessed using a time-structured (blocked/contiguous) split to reduce information leakage due to autocorrelation. Under the same inputs and validation strategy, the GAM outperformed benchmark alternatives (LR, ANN, SVM), confirming that flexible additive modeling is better suited to heterogeneous, weak-signal conditions typical of drainage-outlet PO_4_ time series.

From an application perspective, the proposed method provides a practical decision-support screening tool: it can flag periods of elevated PO_4_ concentration at the outlet to prioritize confirmatory sampling and support evaluation of operational timing (fertilizer/irrigation/drainage) and of downstream protection, while leveraging routinely available measurements in many irrigation districts.

This is an outlet-scale model trained on one catchment over a finite monitoring period; PO_4_ drivers may shift with changes in management, hydrology, or source composition. Accordingly, the approach reduces—but does not replace—laboratory PO_4_ measurements: periodic confirmatory sampling and recalibration are recommended to maintain performance over time and when the approach is transferred to other sites. Future work should extend the approach to longer multi-year records and additional outlets, evaluate updating strategies under regime change, and test whether additional routinely measurable covariates can improve robustness without sacrificing the minimal-input design.

##  Supplemental Information

10.7717/peerj.21181/supp-1 Supplemental Information 1 All parameters used in the model

## Additional Information and Declarations

### Data Availability

The following information was supplied regarding data availability: The raw data is available in the Supplemental File.
